# Correlation Analysis of Protein Expression of 10 HDAC/Sirtuin Isoenzymes with Sensitivities of 23 Anticancer Drugs in 17 Cancer Cell Lines and Potentiation of Drug Activity by Co-Treatment with HDAC Inhibitors

**DOI:** 10.3390/cancers14010187

**Published:** 2021-12-31

**Authors:** Steven Behnisch-Cornwell, Christoph W. Grathwol, Lukas Schulig, Anika Voigt, Daniel Baecker, Andreas Link, Patrick J. Bednarski

**Affiliations:** Department of Pharmaceutical/Medicinal Chemistry, Institute of Pharmacy, University of Greifswald, Friedrich-Ludwig-Jahn-Straße 17, 17489 Greifswald, Germany; steven.behnisch@web.de (S.B.-C.); christoph.grathwol@kit.edu (C.W.G.); lukas.schulig@uni-greifswald.de (L.S.); anika.voigt@uni-greifswald.de (A.V.); daniel.baecker@uni-greifswald.de (D.B.); link@uni-greifswald.de (A.L.)

**Keywords:** anticancer drugs, cisplatin, correlation analysis, histone deacetylases, HDAC inhibitors, lomustine, sirtuins, sirtuin inhibitors, topotecan

## Abstract

**Simple Summary:**

Protein expression profiles of 10 HDAC/Sirtuin isoenzymes in two panels of human cancer cell lines were compared with each other and with the potencies of various anticancer drugs by Pearson and Spearman correlation analysis to identify patterns of enzyme expression and anticancer activity. Furthermore, the NCI COMPARE database was used to identify possible correlations between the mRNA expression in a 60 cancer cell panel and the potency of the same anticancer drugs. While several interesting correlations were found within both data sets, none of these correlations were identical in the two sets of data, suggesting that protein and mRNA expression profiles are not comparable. Combination treatments with several HDAC inhibitors with a number of the anticancer drugs revealed interesting synergistic effects that were in keeping with some of the correlations predicted by our protein expression analysis.

**Abstract:**

Inhibiting the activity of histone deacetylase (HDAC) is an ongoing strategy in anticancer therapy. However, to our knowledge, the relationships between the expression of HDAC proteins and the antitumor drug sensitivity of cancer cells have not been studied until now. In the current work, we investigated the relative expression profiles of 10 HDAC isoenzymes comprising the classes I–III (HDAC1/2/4/6; Sirt1/2/3/5/6/7) in a panel of 17 cancer cell lines, including the breast, cervix, oesophageal, lung, oral squamous, pancreas, as well as urinary bladder carcinoma cells. Correlations between the data of mRNA expression for these enzymes obtained from the National Cancer Institute (NCI) 60 cancer cell line program were also examined. Next, we performed univariate analysis between the expression patterns of HDAC/Sirt isoenzymes with the sensitivity of a 16 cell panel of cancer cell lines towards several antitumor drugs. In a univariate correlation analysis, we found a strong relation between Sirt2 expression and cytotoxicity caused by busulfan, etoposide, and hydroxyurea. Moreover, it was identified that Sirt5 correlates with the effects exerted by oxaliplatin or topotecan, as well as between HDAC4 expression and these two drugs. Correlations between the data of mRNA expression for enzymes with the potencies of the same anticancer agents obtained from the NCI 60 cancer cell line program were also found, but none were the same as those we found with our protein expression data. Additionally, we report here the effects upon combination of the approved HDAC inhibitor vorinostat and one other known inhibitor trichostatin A as well as newer hetero-stilbene and diazeno based sirtuin inhibitors on the potency of cisplatin, lomustine, and topotecan. For these three anticancer drugs, we found a significantly enhanced cytotoxicity when co-incubated with HDAC inhibitors, demonstrating a potentially beneficial influence of HDAC inhibition on anticancer drug treatment.

## 1. Introduction

The epigenetic modulation of protein expression can wield a broad influence on tumor progression. It may cause recurrence of cancer, as well as malfunction of therapy [1,2,3]. Epigenetic regulation is often an effect of post-translational protein modification through processes such as methylation/demethylation or acetylation/deacetylation. The histone proteins are one of the major epigenetic targets responsible for the DNA compaction in the nucleus. Key players of these post-translational modifications are histone deacetylases (HDACs) and acetyltransferases (HATs). The latter enzymes are classified by two groups, dependent on their localization. One type of HAT occurs in the nucleus and acetylates histones there, thus increasing transcription and contributing to gene expression. post-translational acetylation of the side-chain primary amine of lysine residues in histone tails mediated by HATs causes an “open” chromatin conformation, leading to better accessibility of DNA binding sites for transcription factors and thus enhanced RNA polymerase activity in that area. In contrast, HATs of the second type exist in the cytoplasm. These enzymes acetylate histones before forming the nucleosomes [4]. The added acetyl groups can be removed by HDACs following an opposed manner. As epigenetic alterations are reversible, and pharmacological intervention in epigenetic regulation mechanisms represents a contemporary strategy among antineoplastic therapies.

**Figure 1:**
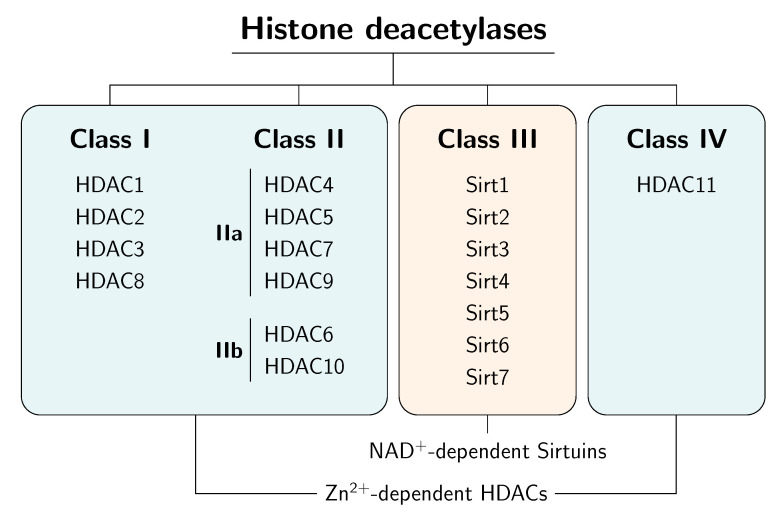
Overview of histone deacetylase classes.

The super-family of HDAC enzymes is subdivided into four classes according to sequence homologies and cofactor dependencies (Figure 1). The classes I, II, and IV mediate hydrolysis activity through a zinc-depending mechanism and are designated as “classical” HDACs. The zinc ion stabilises the acetylated substrate in the catalytic center and polarises the carbonyl group, thereby facilitating the nucleophilic attack of the carbonyl group by a water molecule. The class I HDACs (HDAC1, HDAC2 HDAC3, and HDAC8) have a domain of the yeast transcriptional regulator RPD3 in common and are located in nuclear compartments. The class II HDACs comprise the class IIa (HDAC4, HDAC5, HDAC7, and HDAC9), which shares a large C-terminus, and the class IIb (HDAC6 and HDAC10) contains two acetylase domains. In general, class II HDACs possess only limited enzymatic activity. In the cell, these enzymes migrate between the cytosol and nucleus. HDAC11 is the only member of class IV and exhibits features of both the classes I and II HDACs. However, the specificity of classical HDACs towards single core histones is very low and controversially discussed [5,6,7].

Class III HDACs, the so-called sirtuins (Sirt), distinguish themselves from classical HDACs by their dependency on the cofactor NAD^+^. Interestingly, these enzymes do not use NAD^+^ as a redox active cofactor. Rather, during the enzymatic reaction the acetyl group is transferred to NAD^+^, releasing nicotinamide and a mixture of 2′- and 3′-*O*-acetyl-ADP-ribose (*O*AADPR) as by-products.

In humans, seven sirtuin isoenzymes have been identified (Sirt1–7) and were assigned to four different groups, according to their phylogenetic relationship. Class I includes the isoenzymes Sirt1–3, whereas Sirt4 and Sirt5 are assigned to class II and class III, respectively. The class IV covers the isoenzymes Sirt6–7. The various sirtuins differ in their subcellular localisation, enzymatic activities, as well as substrate specificities. Sirt1, Sirt6, and Sirt7 are usually localised in the nucleus, and Sirt2 is mainly cytosolic. Except for Sirt4 and Sirt5, in vivo deacetylation of core histone proteins is reported for all human sirtuins. Additionally, other acyl groups than acetyl are recognized and cleaved by certain isotypes. In this regard, Sirt5 is associated with the removal of acyl groups derived from dicarboxylic acids (e.g., malonyl, succinyl, and glutaryl) and Sirt6, as well as Sirt2 with the cleavage of long-chain fatty acyl groups, such as myristoyl. Besides their function as epigenetic regulators via histone deacetylation, nearly all HDAC/Sirt isoenzymes possess additional non-histone targets, for example, p53, NF-ϰB, or HIF-1α, which explains their overarching role in apoptosis, cell cycle progression, and ultimately, tumorigenesis [8,9,10].

**Figure 2:**
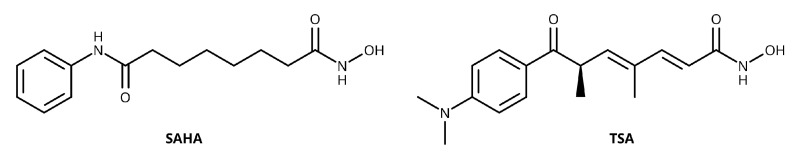
Structures of vorinostat (SAHA) and trichostatin A (TSA).

HDAC inhibitors are currently under investigation because of their promising potential in antineoplastic chemotherapy. Inhibitors of the classical HDAC isotypes can be divided into four groups according to their structural features: (i) hydroxamic acids, such as vorinstat, belinostat, panabinostat, or trichostatin A (for some, see Figure 2), (ii) short chain fatty acids like valproic acid, (iii) 2-aminobenzamides, for example, entinostat, and (iv) cyclic tetrapeptides, such as romidepsin. All these compounds share a common mechanism of action, which is based on their tendency to form highly stable chelate complexes with the HDACs active site zinc ion, ultimately causing loss of enzymatic activity.

In recent years, the HDAC inhibitors vorinostat, panabinostat, belinostat, and romidepsin have been approved as drugs for the treatment of haematological malignancies, as well as various solid tumors [6,7]. Additionally, further HDAC inhibitors, such as valproic acid and roclinostat are currently undergoing clinical trials. Regarding sirtuins, several compounds have been identified as potent inhibitors, even though none of them have yet reached the clinic. Besides analogues of the endogenous pan-sirtuin inhibitor nicotinamide, a few other compounds have been found to inhibit the sirtuin-catalysed deacetylation. These include NAD^+^ mimics, hydroxynaphthaldehyde derivatives, such as sirtinol and cambinol, splitomicins, thiobarbiturates, SirReals, and numerous structurally diverse compounds, such as suramin, tenovin, and aristoforin. The screening of kinase inhibitor libraries has often proven successful in the search for novel sirtuin inhibitors, yielding the Sirt2-selective inhibitor AGK2, for instance. The highly potent and selective Sirt1 inhibitor selisistat (EX 527) represents the most advanced sirtuin-targeting drug candidate and is currently being tested in phase III clinical trials to serve as a disease-modifying agent in Huntington’s disease [10,11,12].

**Figure 3:**
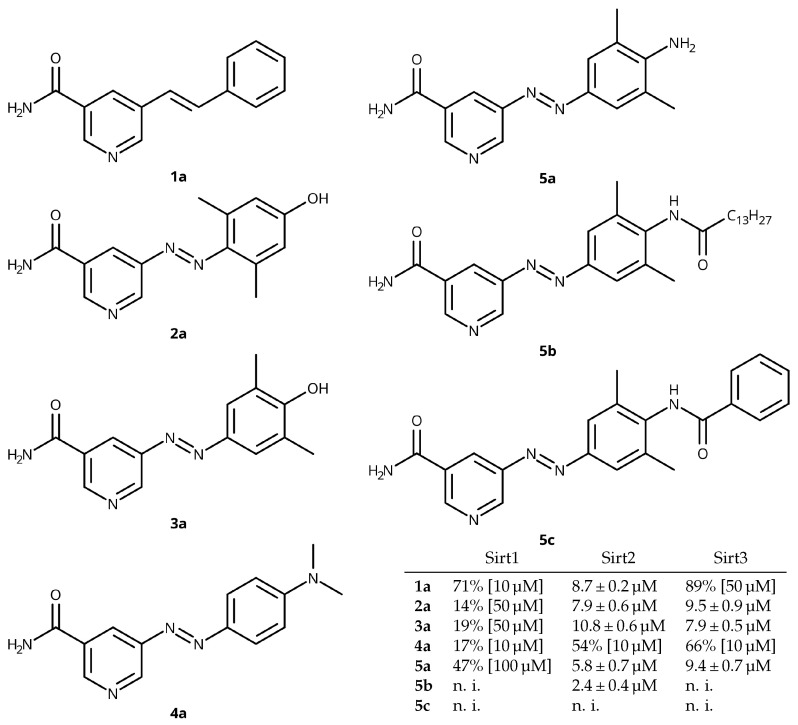
Structures and IC_50_ values in μM or inhibition in % at a fixed concentration of sirtuin inhibitors for Sirt1, Sirt2, and Sirt3, n. i.: no inhibition detected (<30% at 100 μM) [13,14,15,16].

Recently, we reported on the design of photoswitchable sirtuin inhibitors based on the structure of the moderately active, but unselective stilbenoid lead GW435821X. By structural modifications, the bioactivity of the parent compound was increased to the lower micro-molar range and isotype selectivity towards Sirt2 and Sirt3 was improved [13]. Furthermore, replacement of the stilbene C=C-double bond by a diazeno group yielded a series of analogous phenylazopyridines (see Figure 3) that enabled light-mediated modulation of the sirtuin-catalysed deacetylation [14].

Due to the importance of HDAC/Sirt in the epigenomic development of cancer, we asked the question as to how important the expression of specific HDAC/Sirt is on the anticancer action of commonly used chemotherapeutic drugs. Herein, we present a comprehensive study of the HDAC/Sirt expression profiles in a panel of 17 human cancer cell lines from various tumor origins, along with univariate correlation analyses to anticancer drug potency. Furthermore, we investigated the effects of inhibiting the HDAC/Sirt upon combination with selected chemotherapeutics, that is, the approved anticancer drugs cisplatin, lomustine, and topotecan.

## 2. Methods

### 2.1. Chemicals

The compounds **1a**–**5c** were synthesised as recently described [13,14]. All other chemicals were purchased from Sigma Aldrich (Taufkirchen, Germany) except for lomustine, temozolomid, and topotecan, which were obtained from Biomol (Hamburg, Germany). Imatinib mesylate was purchased from Selleckchem (Munich, Germany). The cell culture medium RPMI 1640, as well as penicillin/streptomycin were obtained from PAN Biotech (Aidenbach, Germany), whereas fetal bovine serum was from Sigma Aldrich.

### 2.2. Cell Culture

With one exception, cell lines were obtained from Deutsche Sammlung von Mikroorganismen und Zellkulturen (DSMZ, Braunschweig, Germany); cervical (SISO), breast (MCF-7, EFM-19, MT-3), bladder (RT-4, RT-112, 5637), pancreas (DanG, Pa-Tu-8902, YAPC), lung (A427, EPLC-272H, LCLC-103H), oral (BHY), and esophageal (Kyse-70, Kyse-510, Kyse-520) cancers. The A2780 human ovarian cancer line was provided by Dr. Julie A. Woods, Ninewells Hospital, Dundee, UK. All cells were cultured in RMPI 1640 cell culture medium supplemented with 10% of fetal bovine serum and 1% of penicillin/streptomycin in a humidified atmosphere at 37 °C with 5% CO_2_/95% air. At near confluency, cell cultures were passaged weekly and routinely tested to exclude a potential contamination with mycoplasma. The doubling times of cancer cell lines were determined during the weekly transfer of cells into new cell culture flasks by counting the cells and seeding out a specific cell number to the new flasks. The doubling time was calculated via the following equation, where $t\left(h\right)$ represents the period between the timepoints $t_{0}$ and *t*, which are characterised by their particular cell numbers.

$$\mathrm{doubling}\;\mathrm{time}\;\left(h\right)=\frac{t\left(h\right)}{\log_{2}\left(\frac{\mathrm{cell}\;\mathrm{number}\;t}{\mathrm{cell}\;\mathrm{number}\;t_{0}}\right)}$$

### 2.3. Determination of Antiproliferative Activity (GI_50_ Values)

Fifty percent growth inhibition (GI_50_) was determined by the crystal violet assay, as previously described [17]. In brief, per well of a 96-well plate, 1000 cells were seeded out in 100 μL of culture medium and allowed to attach for 24 h. Subsequently, cells in the exponentially growing phase were exposed to serial dilutions of compounds, added to the medium of the 1000-fold concentrated stock solution in DMSO. After an incubation period of 96 h, cells were fixed with a glutaraldehyde solution (1% in Dulbecco’s buffer) for 20 min. After washing with Dulbecco’s buffer, the cells were stained using a crystal violet solution (0.2% in water) for 30 min. Plates were washed with water and stored for 15 min in water to remove unbound dye. Bound crystal violet was extracted with 70% ethanol for 2 h on a plate shaker, followed by determination of the optical density at λ=570 nm utilizing a SpectraMax Plus 384 plate reader (Molecular Devices, San Jose, CA, USA). The optical density of cells at time point zero of compound exposure (T_0_ control) was subtracted from treated cells and related to untreated control (T/C). The GI_50_ value was calculated with Prism 6.0 Software (Graph Pad, San Diego, CA, USA) by interpolation of the inhibited proliferation at 50%. All GI_50_ values are averages of four or more independent determinations.

### 2.4. Determination of Expression Profiles

The expression profiles of HDAC/Sirt were analysed via the Western blot technique, following the instructions of Biorad (Munich, Germany) and using precast “Criterion TGX Stain-Free Gels” and the corresponding “Trans-Blots Turbo Pack Midi” PVDF membranes. All antibodies were purchased from Cell Signaling Technology (United Kingdom): anti-HDAC1 (#5356), anti-HDAC2 (#5113), anti-HDAC4 (#7628), anti-HDAC6 (#7558), anti-Sirt1 (#9475), anti-Sirt2 (#12650), anti-Sirt3 (#5490), anti-Sirt5 (#8782), anti-Sirt6 (#12486), anti-Sirt7 (#5360), anti-mouse IgG HRP-linked (#7076), and anti-rabbit IgG HRP-linked (#7074).

The culture cells were grown in T_75_ flasks and samples were collected when confluency of 80% was reached and lysed on ice for 30 min with a buffer containing 50 mM Tris (pH 7.4), 100 mM NaCl, 100 mM NaF, 5 mM EDTA, 0.2 mM Na_3_VO_3_, 0.1% Triton-X, and freshly added 1% protease inhibitor cocktail (Sigma Aldrich, Taufkirchen, Germany), followed by a sonication for 10 min. After centrifugation (18,000× *g*, 10 min, 4 °C) the protein concentration was quantified via the Bradford method against bovine serum albumin (BSA) as standard. Protein samples were stored at −80 °C until analysis.

For the electrophoretic separation, 30 μg of total protein were diluted in 22.5 μL of aqua purificata and 7.5 μL of 4× Laemmli buffer containing 0.65 mM 1,4-dithiothreitol, 18.66 μM bromophenol blue, 0.25 mM Tris (pH 6.8), sodium lauryl sulfate 7.5%, and 37.5% glycerol in water. Each slot of the precast gels was loaded with protein samples and blotted onto PVDF membranes after electrophoretic separation. Blots were blocked with 10% non-fat milk powder in Tris buffered saline/tween buffer (TBST) containing 0.02 mM Tris, 0.145 mM NaCl, and Tween 20 (0.5% in water) for 2 h and incubated with primary antibody dilution (1:1000) in TBST plus 1% BSA over night at 4 °C. After washing with TBST, blots were incubated with horseradish peroxidase-conjugated secondary antibody dilution (1:5000) in TBST plus 1% BSA for 2 h at room temperature. Protein bands (Figures S1–S11) were detected with Clarity Western ECL Substrate (Bio-Rad, Feldkirchen, Germany) and recorded with an Advanced Fluorescence Imager (Intas Science Imaging Instruments, Göttingen, Germany).

The used TGX Stain-Free Gels include unique trihalo compounds that allow a rapid fluorescent detection of proteins. The trihalo compounds react with tryptophan residues in a UV-induced reaction to produce fluorescence. The fluorophores remain covalently bound to the proteins and were detected after the blotting procedure by a Gel Doc EZ imager (Bio-Rad, Feldkirchen, Germany). The use of stain-free imaging allows the normalisation of bands to the total protein on a blot, eliminating the use of housekeeping proteins, such as β-actin or GAPDH [18,19]. The total protein served as an internal control used with the TGX Stain-Free Gels systems from Bio-Rad.

The band intensities of the target proteins were related to the signal of the corresponding band column on the stain-free image. The relative protein expression was calculated by the corrected band intensity of the target protein related to the mean band intensity over all cell lines. All results are averages of four or more independent Western blot determinations.

### 2.5. Data from the National Cancer Institute 60 Cell Line Program

The data provided by the National Cancer Institute (NCI) were downloaded from dtp.cancer.gov, https://dtp.cancer.gov/mtweb/search.jsp, accessed on 15 May 2020. For the expression of mRNA, the following experiment ID numbers were used: HDAC1 #GC29185, HDAC2 #GC15516, HDAC4 #GC28681, HDAC6 #GC12671, Sirt1 #GC64431, Sirt2 #GC16536, Sirt3 #GC91754, Sirt5 #GC13785, Sirt6 #GC78925, and Sirt7 #GC46856.

### 2.6. Statistics

For statistical evaluation and visualisation, the Python packages statsmodels v0.11.1 [20], Matplotlib v.3.1.3 [21], and Prism v6.0 Software (Graph Pad, San Diego, CA, USA) were used. The Pearson and Spearman correlation coefficients were used to assess the level of significance, referring to similar studies as previously published [17,22]. Pearson correlation coefficients *R* were calculated by using ordinary least squares (OLS) and false discovery rate (FDR) corrections using the Benjamini-Yekutieli procedure [23]. The corresponding plots with multiple testing comparisons are available in the Supporting Information (Figures S16–S19). For the statistical analysis of the influence caused by the HDAC inhibitors on anticancer drug potency (Section 3.4), an ordinary one-way ANOVA was performed with Dunnett’s multiple comparison test adaption against the control. In general, the mean ± standard deviation (SD) is given and the level of significance is expressed as * p<0.05, ** p<0.01, *** p<0.001, and **** p<0.0001.

## 3. Results

### 3.1. Expression of HDAC/Sirt Isoenzymes and Correlation Analysis

**Figure 4:**
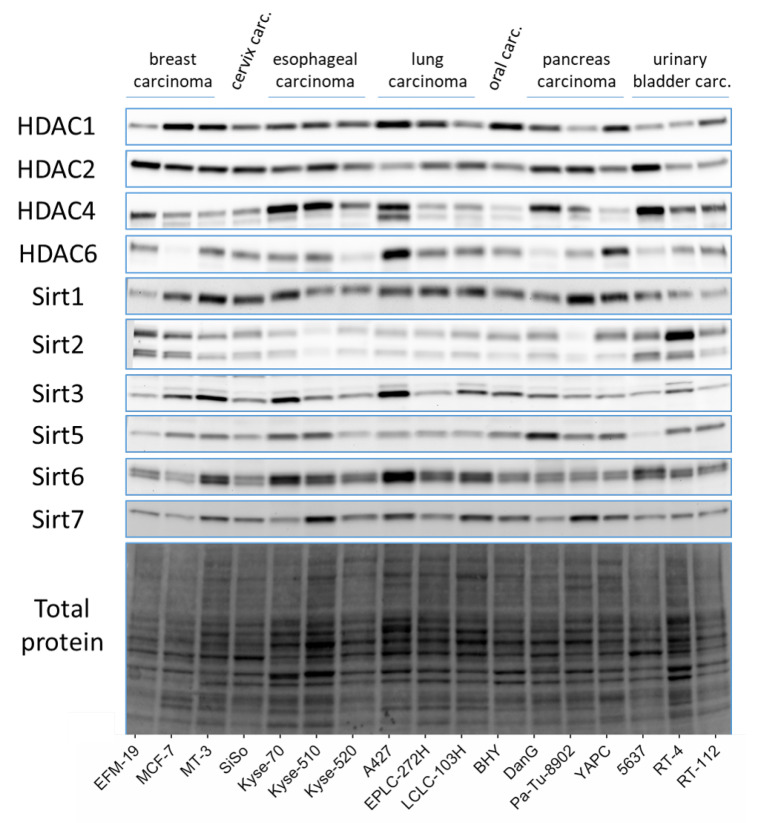
Representative Western blots of protein expression for HDAC/Sirt isoenzymes in 17 human cancer cell lines.

**Figure 5:**
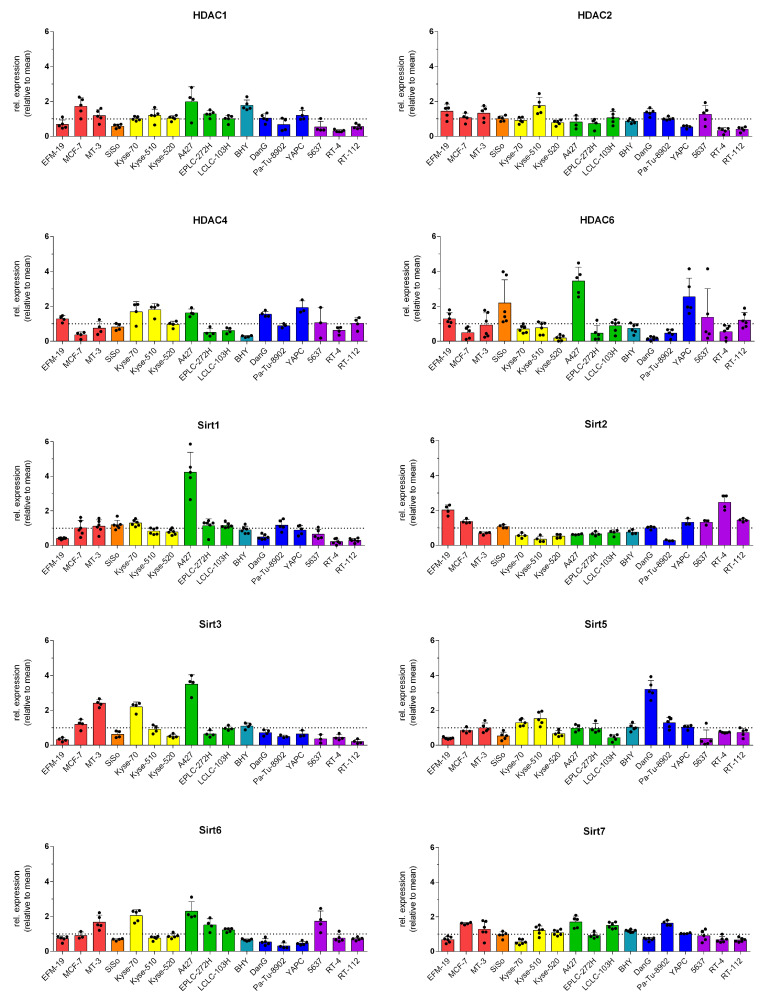
Relative protein expression profiles of HDAC/Sirt isoenzymes in 17 human cancer cell lines (relative to the mean expression over all cell lines) [mean ± SD of n≥ three independent determinations; breast carcinoma (red), cervix carcinoma (orange), oesophageal carcinoma (yellow), lung carcinoma (green), oral squamous carcinoma (cyan), pancreas carcinoma (blue), and urinary bladder carcinoma (purple)].

A representative set of corresponding Western blots showing the expression of the HDAC/Sirt in a panel of 17 human cancer cell lines is pictured in Figure 4 and the relative protein expression profiles are compiled in Figure 5. Table S1 contains the underlying dataset. The HDAC1 isoenzyme is most pronounced in the cell lines MCF-7, BHY, and A427, whereas the urinary bladder cancer cell lines 5637, RT-4, and RT-112 show the lowest expression. Among the tested cell lines, the profile of HDAC2 is less varying than for HDAC1 as the relative expression ranges between 0.7 and 1.3 for most cell lines. Compared to the mean, only YAPC, RT-4, and RT-112 possess lower levels, whereas EFM-19 and Kyse-510 cells show higher content. Regarding HDAC4, the greatest expression was found in DanG, YAPC, A427, Kyse-70, and Kyse-510, while BHY, MCF-7, and LCLC-103H cells had the lowest. In the case of HDAC6, the relative expression of protein varies much more compared to the other HDAC isoenzymes. The highest relative levels of 3.45-, 2.55-, and 2.19-fold of the mean were found in A427, YAPC, and SiSo cells, respectively. However, the lowest quantities were detected in DanG (0.16), Kyse-520 (0.19), and Pa-Tu-8902 (0.46).

For Sirt1, the highest expression was identified in A427 cells, whereas EFM-19, DanG, 5637, RT-4, and RT-112 cells exhibited low relative protein expression with values less than or equal to 0.65. The total Sirt2 protein consists of two isoforms, one with 43 kDa and a smaller variant with 39 kDa. For the determination of the relative expression profile, both isoforms were used in sum. The greatest level of Sirt2 was detected in RT-4 and EFM-19 cell lines, and the lowest level was determined in Pa-Tu-8902 cells. Noticeably, all of the oesophageal carcinoma and lung carcinoma cell lines expressed less Sirt2 than the mean of all tested cell lines. A427, MT-3, and Kyse-70 cells had the highest content of Sirt3, whereas RT-112, EFM-19, and 5637 cells exhibited the lowest expression. For Sirt5, we found low levels of protein in 5637 cells, followed by EFM-19, SiSo, and LCLC-103H cells with the same range of protein expression. The highest amount of Sirt5 was detected in DanG cells, then in Kyse-510 and Kyse-70. For the Sirt6 levels, also the sum of two isoforms (36 and 39.1 kDa), the levels fluctuated notably in the tested cell lines with the highest expression in A427 cells, followed by Kyse-70 cells with values greater than 2.0 [24]. The pancreas carcinoma cell lines Pa-Tu-8902, YAPC, and DanG had the weakest expression of Sirt6, with values below 0.54. Regarding Sirt7, we detected values between 0.55 for Kyse-70 cells and 1.71 for A427 cells. In relation to all sirtuin isotypes, Sirt7 was most equally balanced among the tested cell lines.

The lung carcinoma cell line A427 is distinguished in that it exhibits greater expression of HDAC/Sirt isoenzymes compared to the other cancer cell lines included in this analysis. However, the profile of the cervix carcinoma cell line SiSo was most balanced considering the variance among the other cells.

**Figure 6:**
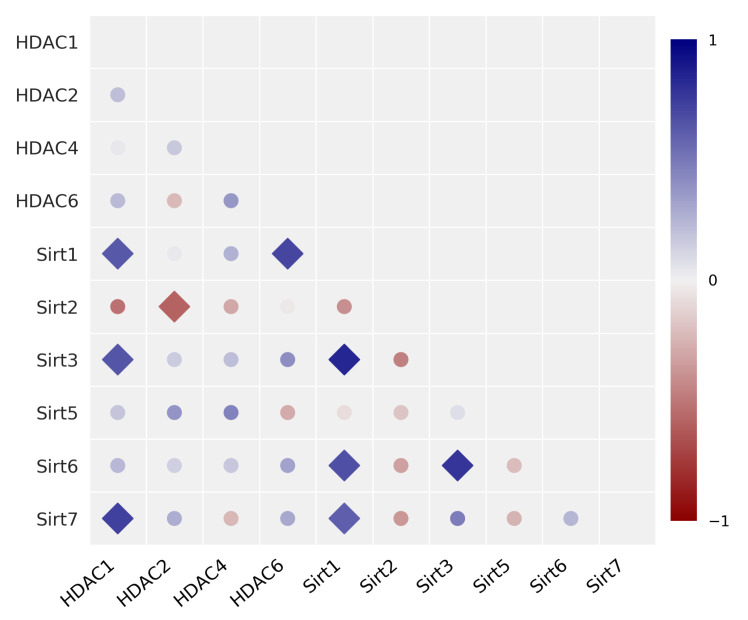
Univariate correlation matrix (Pearson correlation coefficient) for the expression of HDAC/Sirt isoenzyme proteins in 17 human cancer cell lines [statistics: ⧫p<0.05, • not significant]. Positive correlations are depicted in blue, and negative in red (see scale-bar).

Pearson-*R* correlation analysis was first performed to evaluate potential correlations between the protein expression of various HDAC/Sirt isoenzymes. Figure 6 illustrates the results in a correlation matrix, whereas the corresponding *R* and *p*-values are compiled in Table S2. No correlations were detected among HDAC isoenzymes of class I and II, in particular, HDAC1, 2, 4, and 6. However, a clearly positive and significant correlation was detected between HDAC1 and HDAC6 with Sirt1, as well as HDAC1 with the expression of Sirt3 and Sirt7. We discovered a significant inverse correlation between HDAC2 and Sirt2. Within the group of sirtuins, significant positive correlations were found among each other for Sirt1 with Sirt3, Sirt6, and Sirt7, as well as Sirt6 and Sirt3, and Sirt2 and Sirt3 (inverse).

Moreover, we performed a Spearman correlation analysis to identify possible trends in the rankings of the various HDAC/Sirt isoenzymes. The correlation matrix considering the Spearman correlation coefficients is depicted in Figure S12 and the related data (*R* and *p*-values) are given in Table S3. Consistent with the results from the Pearson analysis, no correlations exist among the isoenzymes HDAC1, 2, 4, and 6. Spearman analysis also revealed positive significant correlations between HDAC1 with the expression of Sirt3 and Sirt7, and Sirt1 with Sirt3. Additionally, the correlation of Sirt3 with Sirt7 was found significant when performing the Spearman analysis.

### 3.2. Correlation Analysis with Data from the NCI 60 Cancer Cell Line Program

**Figure 7:**
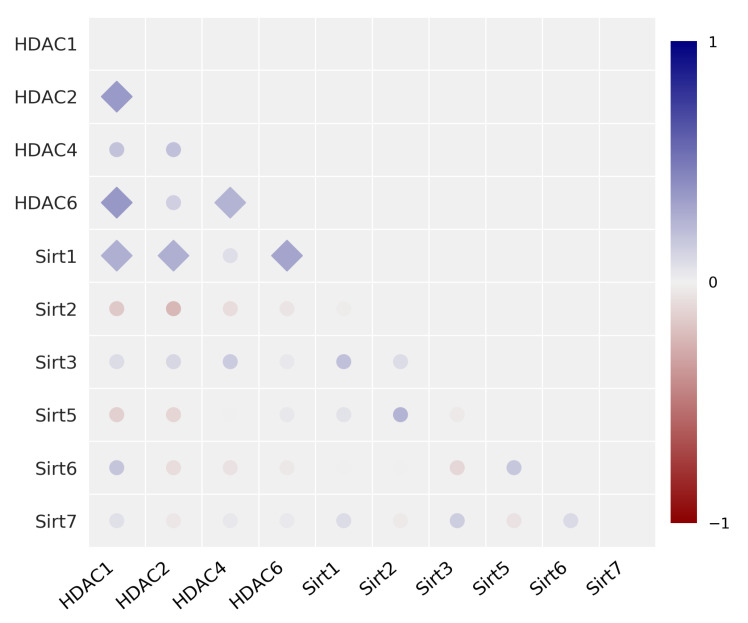
Univariate correlation matrix (Pearson correlation coefficient) for the expression of HDAC/Sirt isoenzyme mRNA with data of NCI 60 cancer cell line program [statistics: ⧫p<0.05, • not significant]. Positive correlations are depicted in blue, and negative in red (see scale-bar).

Using the data available from the NCI 60 cancer cell line program, we performed analogous correlation analysis with the data of mRNA expression for the corresponding HDAC/Sirt isoenzymes. The NCI ID numbers of the adduced enzymes are listed in the Methods section. The univariate Pearson correlation matrix of mRNA expression is shown in Figure 7 and the corresponding data values are compiled in Table S4. The correlation matrix considering the Spearman correlation coefficients is depicted in Figure S13. In the comparison of the mRNA expression data from 60 cell lines, positive correlations of HDAC1 mRNA expression were found between HDAC2 and HDAC6, with significant *R*-values of 0.354 and 0.364, respectively, and between HDAC4 and HDAC6 (0.258). There were also correlations between Sirt1 with HDAC1 and HDAC2 (0.269), as well as with HDAC6 (0.316). Further significant correlations between the mRNA expression of the isoenzymes were not identified. However, as mentioned above, analysing the correlation of the protein expression seems to better represent the epigenomic functionality as compared to the data from the NCI mRNA analysis. Therefore, the lack of revealing outcomes when screening the NCI mRNA 60 cell line database is not too surprising. Nevertheless, the correlations we identified for Sirt1/HDAC1 and Sirt1/HDAC6 are in agreement with those of NCI.

### 3.3. Antiproliferative Activity of SAHA and TSA—Correlations with HDAC/Sirt Expression

To evaluate whether the antiproliferative activity of the HDAC inhibitors SAHA and TSA correlates with the expression of zinc dependent HDAC isoenzymes, the potency of both compounds in a panel of 16 human cancer cell lines was determined, expressed as 50% growth inhibitory values (GI_50_).The 16 cell line panel differed from the previous 17 cell line panel by the removal of the EFM-19 and EPLC-272H cell lines and the addition of the A2780 human ovarian cancer cell line. Table 1 shows the determined GI_50_ values, as well as the relative GI_50_ (GI_50_ value related to the mean over all tested cell lines).

**Figure 8:**
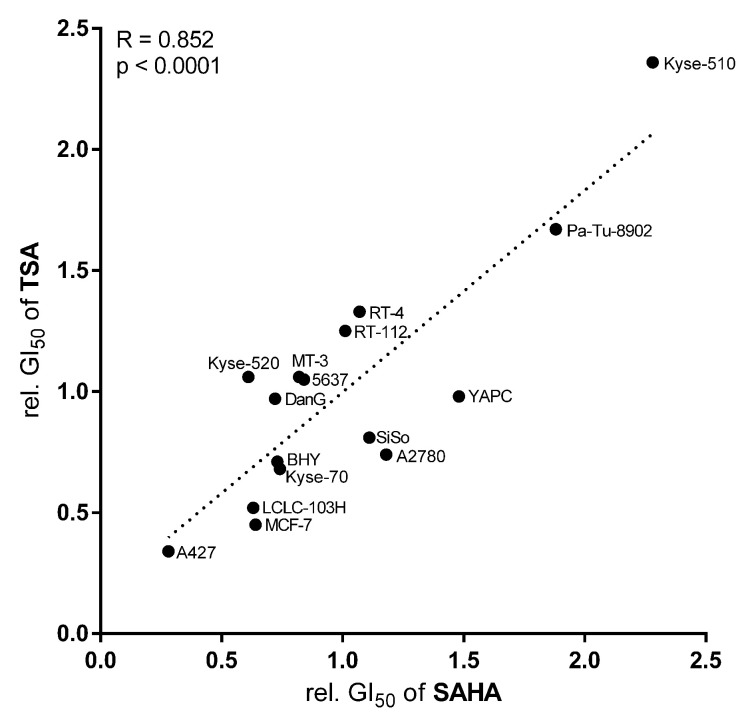
Univariate correlation analysis of SAHA with TSA cytotoxic potency expressed as relative GI_50_ values (relative to the mean GI_50_ over all cell lines) in 16 human cancer cell lines [R=0.852, p<0.0001].

For SAHA, the GI_50_ values were in the single-digit micromolar scale, whereas the GI_50_ values for TSA were approximately ten-fold lower. To assess the likelihood that SAHA and TSA have the same mechanism of action, a correlation analysis was performed with the relative GI_50_ values for TSA and SAHA across the 17 cancer cell lines (Table 1). Similar correlation analyses were used in the NCI COMPARE program to identify substances with similar mechanisms of anticancer action [25]. The results in Figure 8 show a highly significant correlation between the potency of both compounds in the cancer cell lines, with an *R*-value of 0.852 (p<0.0001). The most sensitive cell line towards zinc dependent HDAC inhibitors was A427 followed by MCF-7 and LCLC-103H. The cell line A427 already revealed the strongest HDAC/Sirt expression among the cell lines (Table 1). In contrast, the cell lines Kyse-510 and Pa-Tu-8902 were the least sensitive.

We hypothesise that the anticancer activity of SAHA and TSA could correlate with the expression of specific zinc-dependent HDAC isoenzymes. To investigate this hypothesis, a Pearson correlation analysis was performed with the GI_50_ values of either SAHA or TSA in 16 cell lines and the corresponding relative protein expression of zinc-dependent HDAC isoenzymes. However, no significant correlations were found between these variables, with *R*-values only ranging between −0.44 and 0.30.

To investigate whether there is a connection between the expression of HDAC/Sirt proteins and the cytotoxic effect of six tested anticancer drugs (Table 2), a univariate correlation analysis was performed with the relative GI_50_ values of the antitumor drugs of each cell line to the corresponding relative protein expression of the HDAC/Sirt isoenzyme. The GI_50_ values of most of the anticancer drugs in 14 of the cell lines were taken from our earlier investigations [17], while the GI_50_ values of imatinib, lomustine, temozolomide, bortezomib, and topotecan were determined within the scope of the present study in Table 2.

In the case of imatinib, the cell lines A427 and DanG were quite sensitive with similar GI_50_ values of 6.5 ± 5.6 μM and 6.8 ± 1.2 μM, respectively. The highest GI_50_ was found in Pa-Tu-8902 with a value of 16.6 ± 0.7 μM. For lomustine, the most sensitive cell line was MT-3 with a GI_50_ of 3.5 ± 0.4 μM. The average GI_50_ of lomustine in every cell line was 28.3 μM. Relative to the mean GI_50_, the GI_50_ in MT-3 cells is eight times lower, documenting the sensitivity of this cell line to lomustine. In the case of temozolomide, the investigations revealed that the GI_50_ value was greater than 400 μM for most of the studied cell lines. However, only A427 showed a low GI_50_ value of around 6.1 μM, followed by Kyse-510 (22.8 μM), LCLC-103H (71.3 μM) and Pa-Tu-8902 cells (211.9 μM).

The GI_50_ values of bortezomib ranged from 4.1 nM (Pa-Tu-8902) to 9.0 nM (Kyse-510) in the 11 cell lines. The potency of paclitaxel was unspecific, with GI_50_ values between 1.0 and 1.7 nM, except for MT-3 cells, where an increased GI_50_ value of 3.6 nM was found. In contrast, the antiproliferative potency of topotecan varies much more between the cell lines, with an about 5-fold difference between the most sensitive cell line MT-3 (10.2 ± 4.2 nM) and the least susceptible Kyse-510 (56.3 ± 1.4 nM).

**Figure 9:**
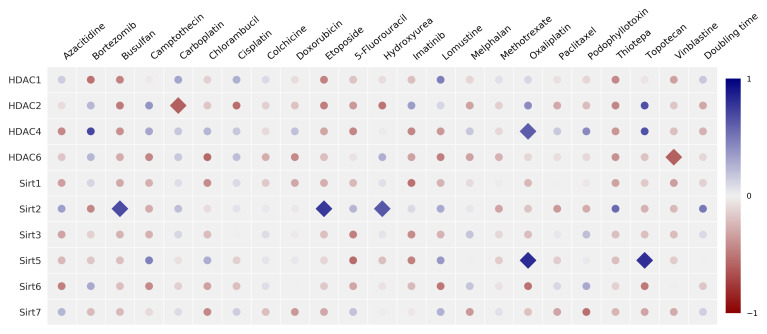
Univariate correlation matrix (Pearson correlation coefficient) concerning the expression of the HDAC/Sirt isoenzyme protein with anticancer drug potency expressed as GI_50_ values and the doubling time of cancer cells [statistics: ⧫p<0.05, • not significant]. Positive correlations are depicted in blue, negative in red (see scale-bar).

The matrix for the correlation of the relative protein expression and the corresponding anticancer drug potency (expressed as GI_50_ value) is shown in Figure 9, with the corresponding *R* and *p*-values compiled in Table S6. A positive correlation means that high protein expression is associated with high GI_50_ values (direct correlation), whereas a negative relation (inverse correlation) implies that high protein levels are accompanied by low GI_50_ values (greater potency).

For the zinc-dependent HDAC2 isoenzyme, a significant correlation (p<0.05) was found for carboplatin. For HDAC4, a highly significant positive correlation was detected with oxaliplatin, as well as a non-significant correlation with topotecan and bortezomib and a negative correlation for HDAC6 with chlorambucil and vinblastine.

Significant relations were also found between the expression of the NAD^+^-dependent Sirt enzymes and anticancer drug potency. A positive significant correlation was indicated between Sirt2 expression and potency mediated by busulfan, etoposide, and hydroxyurea, whereas no significance was found regarding the potency caused by thiotepa. There is a positive correlation (p<0.05) between the expression of Sirt5 with resistance to oxaliplatin and topotecan.

### 3.4. Correlation of Anticancer Drug Potency and the Doubling Time of Cancer Cells with HDAC/Sirt Expression

Additionally, correlation analyses were done to detect a potential connection of the doubling time of cancer cells with the HDAC isoenzyme expression. The calculated doubling times of the cancer cell lines are compiled in Table S5. The Pearson correlation analysis (data shown in Figure 9 and Table S6) rules out a linear correlation between the expressions of various HDAC/Sirt isoforms with the doubling time. Likewise, no significant correlation was found when performing the Spearman analysis. The corresponding correlation matrix of the Spearman correlation coefficients is shown in Figure S14 and the respective data are compiled in Table S3.

### 3.5. Correlation Analysis of Anticancer Drugs Potency and the mRNA Expression with Data from the NCI 60 Cancer Cell Line Program

**Figure 10:**
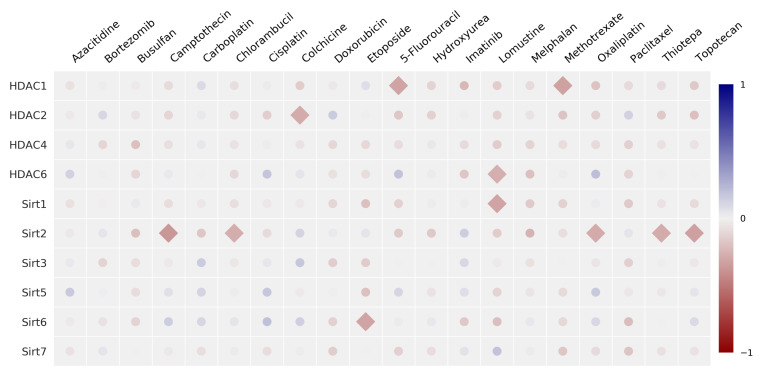
Univariate correlation matrix (Pearson correlation coefficient) with data from the NCI 60 cancer cell line panel for the expression of HDAC/Sirt isoenzyme mRNA with anticancer drug potency expressed as GI_50_ values of cancer cells [statistics: ⧫p<0.05, • not significant]. Positive correlations are depicted in blue, negative in red (see scale-bar).

Analogous correlation analysis of HDAC isoenzyme mRNA expression and the potency of anticancer drugs was performed with data from the NCI 60 cancer cell line program (dtp.cancer.gov, https://dtp.cancer.gov/mtweb/search.jsp, accessed on 15 May 2020). The univariate correlation matrix of mRNA expression is shown in Figure 10, and the corresponding values in Table S7. In total, the data from 60 cell lines were compared. A few significant inverse correlations were found, however, the *R*-values were below −0.370 and only representatives of inverse correlations showed significance, while the positive ones were not statistically significant. However, the very large number of cell lines still makes such correlations significant. Remarkably, only inverse correlations were detected between enzyme mRNA expression and GI_50_ values—that is, mRNA expression of HDAC1 with 5-fluorouracil and methotrexate, HDAC2 with colchicine, HDAC6 and Sirt1 with lomustine, Sirt2 with camptothecin, chlorambucil, oxaliplatin, thiotepa, and topotecan and between Sirt6 with etoposide. This is in contrast to our data in the 17 cancer cell line panel with protein expression, where both negative and positive correlations were found. The corresponding correlation matrix considering the Spearman correlation coefficients is given in Figure S15 and the data set in Table S4. Only mRNA expression of Sirt1 showed a positive, significant correlation with bortezomib.

### 3.6. Combination Effects of HDAC Inhibition on Anticancer Drug Potency

The stilbenoid derivative **1a** and the other diazeno-based sirtuin inhibitors **2a**–**5c** did not exhibit activity in the tested cell lines up to 100 μM. Only in case of **2a** were GI_50_ values in the low micromolar range; that is, 4.6 ± 2.1 μM (5637), 10.8 ± 0.4 μM (SiSo), 8.8 ± 1.7 μM (Kyse-70), 5.4 ± 1.5 μM (RT-4), and 7.1 ± 4.1 μM (RT-112). Due to the low cytotoxicity of the sirtuin inhibitors a correlation analysis according to Section 3.3 and Section 3.4 was not performed for these compounds.

To assess whether HDAC inhibitors can affect the potency of the anticancer drugs in combination treatments, the SiSo human cervical cancer cell line was chosen for further experimentation because of a balanced protein expression among all analyzed HDAC/Sirt isoenzymes (Figure 5). SiSo cells were exposed to inhibitors at fixed, non-toxic concentrations. For these studies, a concentration was selected that showed a maximal inhibition of proliferation by 15%, as deduced from the dose-response curve (SAHA: 0.3 μM, TSA: 0.03 μM, **2a**: 5 μM, **1a** and **3a**–**5c**: 50 μM). The HDAC/Sirt inhibitors were tested in combination with serial dilutions of the anticancer drugs cisplatin, lomustine, and topotecan. Cisplatin and topotecan were chosen because these are commonly used drugs to treat cervical cancer while the potency of lomustine has been reported to increase in glioblastoma cell lines when combined with TSA [26]. The GI_50_ values for the inhibition of proliferation were determined. The combination effect is expressed as relative combination growth inhibition at 50% (CGI), which is the value of the quotient of the GI_50_ in combination (drug + the HDAC inhibitor) divided by the control GI_50_ (drug alone). A CGI<1 indicates enhanced anticancer drug potency as a result of the HDAC inhibitor, a CGI>1 indicates diminished potency and with CGI=1 the HDAC inhibitor has no effect on the potency of the anticancer drug.

**Figure 11:**
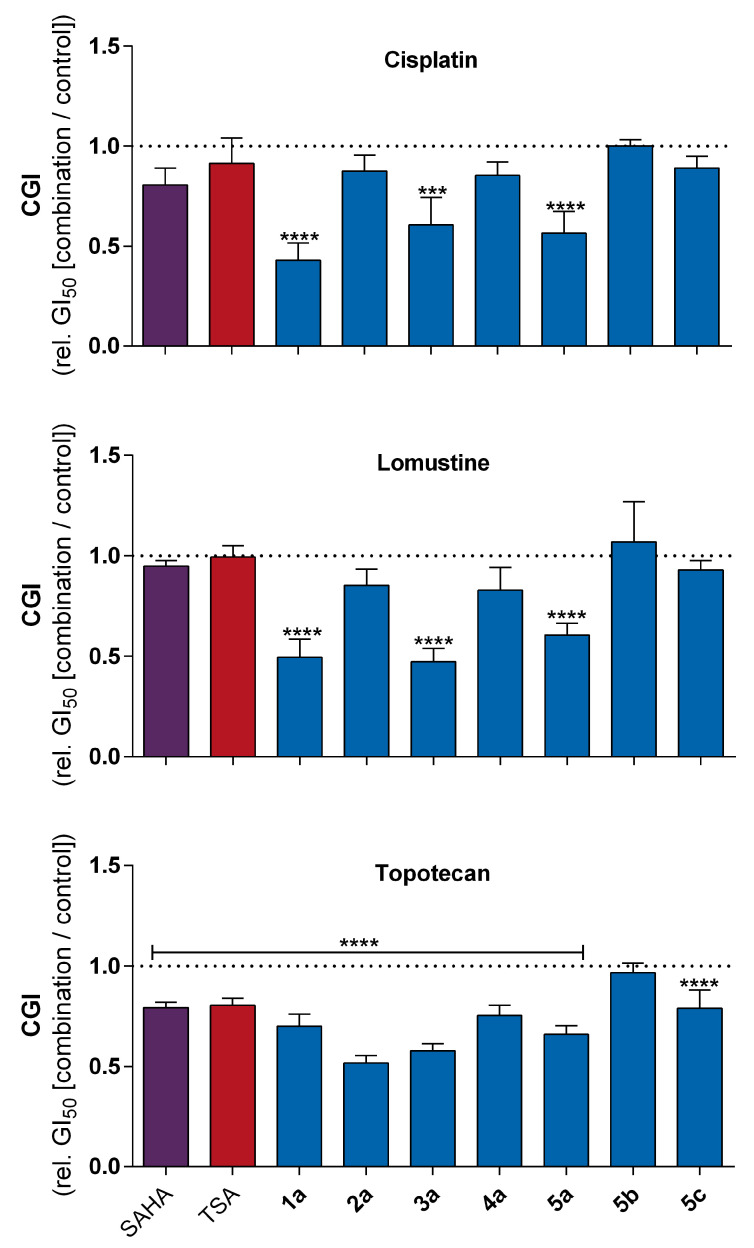
Relative GI_50_ values of cisplatin, lomustine, and topotecan in combination with HDAC inhibitors (SAHA: 0.3 μM, TSA: 0.03 μM, **2a**: 5 μM, **1a** and **3a**–**5c**: 50 μM) related to the GI_50_ of the respective anticancer drug without inhibitor (CGI) in the SiSo cell line; dotted lines mark the control without inhibitor incubation [mean + SD of n>3; statistics: one-way ANOVA, Dunnett’s multiple comparison test; *** p<0.001, **** p<0.0001].

Figure 11 shows the determined CGI for combinations of various HDAC inhibitors with the anticancer drugs in the SiSo cell line, while the raw GI_50_ and CGI values are shown in Table 3. The combination of cisplatin with several HDAC inhibitors resulted in an enhanced potency of cisplatin in some cases. For instance, the combination of cisplatin with SAHA and TSA showed just a slightly decreased GI_50_ with CGI values of 0.81 and 0.91, respectively. Similar results were obtained in combinations with **2a** (0.88), **4a** (0.86), and **5c** (0.89). For the combination of cisplatin with **1a**, **3a**, and **5a**, a significantly enhanced potency of cisplatin was detected, with decreased GI_50_ values from 238 to 135, 190, and 121 nM, corresponding to CGI values of 0.43, 0.61, and 0.57, respectively.

The potency of lomustine was not enhanced by either SAHA or TSA. As with cisplatin, the GI_50_ of lomustine decreased significantly in combination with **1a**, **3a**, and **5a** to values of 2.4, 2.3, and 2.9 μM, and with CGI values of 0.50, 0.47, and 0.59, respectively. Combining with **2a** and **4a**, the CGI were only slightly decreased (0.85 and 0.83) and left unaffected in the case of **5b** and **5c** (1.07 and 0.93).

The potency of topotecan was significantly enhanced in combination with SAHA and TSA, resulting in decreased GI_50_ of topotecan from 20.2 to 16.3 and 16.5 nM, with CGI values of 0.63 and 0.80, respectively. The combination with the stilbenoid derivative **1a** or the diazeno-based sirtuin inhibitors with the exception of **5b** led to a significantly enhanced potency of topotecan. The GI_50_ value of topotecan decreased significantly, in some cases by half, to 13.5 (**1a**), 10.0 (**2a**), 11.2 (**3a**), 14.6 (**4a**), 12.8 (**5a**), and 15.4 nM (**5c**) upon co-incubation. Corresponding CGI values ranged from 0.52 to 0.79.

## 4. Discussion

In our previous studies, we sought associations between the antiproliferative activity of 19 anticancer drugs with the activity of various antioxidative enzymes in a similar panel of human cancer cell lines [22]. In the present work, correlations between the expression profiles of HDAC/Sirt in various cancer cell lines and the potency of anticancer drug potency were investigated.

HDACs represent a family of key epigenetic modulators. It has been reported that the expression of zinc dependent HDACs can be significantly increased in neoplastic compared to healthy tissues [27,28]. However, expression of sirtuins in cancer would seem to be much more complex. Various studies report an upregulation of sirtuins in cancer cells, while others demonstrate a downregulation in comparison to normal tissues [29]. Thus, the role of sirtuins in cancer cells may, on the other hand, be double-edged because of tumor suppression and oncogenic properties.

For our studies, we expected an irregular protein expression of HDAC isoenzymes across the tested cell lines and assumed that a low protein expression of a certain isoenzyme could be compensated by an increase in the expression of another isoenzyme, reflected by a significant inverse correlation (negative correlation). However, no evidence was found to confirm this hypothesis. Except for HDAC2 with Sirt2, no such negative correlations were apparent. Instead, a few positive correlations were detected. Positive correlations were found between HDAC1 with Sirt1, Sirt3 and Sirt7, HDAC6 with Sirt1, Sirt1 with Sirt3, Sirt6, and Sirt7, as well as Sirt6 with Sirt3. The genes for the HDAC/Sirt isoenzymes are spread widely across the chromosomes; an overview of the localisation is given in Table S8. This fact rules out that significant positive correlations are due to adjacent or closely localized genes on the same chromosome. To the best of our knowledge, there are no comparable studies in the literature that report similar findings.

Analogous correlation analyses were performed with data from the NCI 60 cancer cell line program. The NCI collects data of mRNA expression for genes as well as antiproliferative potencies (GI_50_) of a large number of small molecules such as anticancer drugs in a panel of 60 various cancer cells lines of differing tumor origin. Compiled datasets are accessible free of charge to the public by an Internet-based data bank. On the other hand, data with protein expression profiles are not available, probably because of the high expense and effort such protein analysis by Western blotting would entail. Here, we have performed correlation analysis with protein expression, which we believe more accurately mirrors the cellular epigenomic functionality comparable to data obtained from mRNA analysis. Interestingly, none of the correlations we found between the HDAC/Sirt enzyme expression in our 17 cancer cell line panel were found in the corresponding NCI data with 60 cancer cell lines, and *vice versa*. Likewise, none of the correlations we found between HDAC/Sirt enzyme expression and anticancer drug potencies in the 17 cell line panel were present in the corresponding NCI data, and *vice versa*. While the two panels of cancer cell lines have different sizes and different cancer cell lines, in a previous study where we compared relative potencies of anticancer drugs in these two panels, many of the same correlations were confirmed in both panels [15]. On the other hand, when we compared the relative enzyme activities of various antioxidative enzymes with the corresponding mRNA expression in the 60 cell NCI panel, no apparent correlations were found [22]. Thus, we believe that the reason for these discrepancies in the present study are due to differences in comparing protein expression with data reporting on mRNA expression.

Further analysis of the protein expression of the HDAC/Sirt isoenzymes in relation to the potency of several anticancer drugs revealed numerous correlations. For the anticancer drugs busulfan and hydroxyurea, significant positive correlations with Sirt2 levels were found. As a result, cancer cells that possessed lower levels of Sirt2 protein were more sensitive to the cytotoxic effects of the respective drugs and *vice versa*. In the literature, a connection between Sirt2 protein expression and busulfan, as well as hydroxyurea potency has not yet been described. Additional studies need to be conducted to confirm this finding. Moreover, no significant positive correlation between the toxicity mediated by 5-fluorouracil and expression of Sirt2 was detected. Combination studies of unspecific zinc dependent HDAC inhibitors as well as sirtuin inhibitors have been performed by several groups, who found a positive combination effect between 5-fluorouracil and various HDACs [30,31]. Similarly, significant positive correlations were found between the topoisomerase inhibitor etoposide and Sirt2 protein expression with Sirt5, and an inverse correlation of the vinca alkaloid vinblastine with HDAC6. While the beneficial effect of a combination of class I HDAC inhibitors with etoposide, topotecan, and vinca alkaloids has already been reported [32,33,34], a correlation between these drugs and Sirt2 protein expression is new.

Interestingly, Grohmann et al. showed that the inhibition of nicotinamide phosphoribosyltransferase, which causes leakage of NAD^+^, leads to an increased sensitivity of cells towards etoposide via a Sirt2-dependent mechanism [35]. Likewise, Hoffmann et al. showed that the potency of etoposide is increased due to the inhibition of Sirt2 [36].

The connection between HDAC6 functionality and vinca alkaloid activity was shown by Tu et al. [37] Inhibition of HDAC isoenzymes by SAHA or the HDAC6-specific inhibitor MPT0G211 has been reported to lead to increased vinblastine toxicity [37,38].

For the platinum complexes cisplatin and carboplatin, an inverse correlation with the expression of HDAC2 protein was found, while the potency of oxaliplatin inversely correlates with the expression of the Sirt6 protein and with HDAC4 and Sirt5 in a positive manner. Several studies have shown that various HDAC inhibitors such as SAHA and other hydroxamic acid derivatives increase cisplatin toxicity in cancer cells or can circumvent cisplatin resistance [39,40,41,42,43], supporting the association between the activity of cisplatin or carboplatin and the expression of HDAC2. Interestingly, for another clinically used platinum complex, oxaliplatin, a reverse correlation with the Sirt6 expression (not significant), as well as a significant positive correlation with HDAC4 and Sirt5 expression were found. Analogous investigations of correlations between anticancer drug potency and mRNA expression of HDACs and Sirts with data from the NCI 60 cancer cell program were not in accordance with our results. We believe that our findings are especially relevant because protein expression more closely characterises the epigenomic functionality of cancer cells. Importantly, on the basis of the current study, new insights have been gained into the relationships between HDAC protein expression and anticancer drug sensitivity, which should be useful for investigating possible drug combination therapies.

Another goal of our work was to seek possible connections between the potency of the unselective zinc depending HDAC inhibitors SAHA and TSA and the expression profiles of class I and II HDAC isoenzymes. In the first step, the antiproliferative activity of both was determined in a panel of cell lines. Our data suggested a strong correlation between their activity profiles, revealing the same mode of action in our panel of cancer cells. However, no connection between the expression profiles of HDAC and the SAHA and TSA potency was detected in the univariate correlation analysis. One interpretation is that the toxicity of both drugs is not primarily associated with an inhibition of HDACs. It is known that SAHA and TSA induce apoptosis [44,45,46]. Moreover, it was already described in the literature that SAHA and TSA cause their antitumor effects by addressing non-histone targets [47,48,49,50].

Besides the correlation analysis of HDAC protein expression and anticancer drug potency, we performed combination cytotoxicity testing of HDAC inhibitors with several anticancer drugs. These studies focussed on the effect of SAHA and TSA as well as the newer sirtuin inhibitors on the potency of cisplatin, lomustine, and topotecan. For the combination analysis, the GI_50_ values of the anticancer drugs were determined in the presence and absence of non-toxic concentrations of the HDAC inhibitors. Due to the high cytotoxicity of SAHA and TSA, the concentrations used were quite low, that is, 0.30 and 0.03 μM, respectively. For combinations with lomustine, no effect on anticancer drug potency was observed. In contrast, Staberg et al. reported an increase in the potency of lomustine with the co-treatment of TSA in glioblastoma cell lines [26]. As mentioned previously, it is known that SAHA as well as TSA can increase the potency of cisplatin. Our combination studies of the hydroxamic acid derivative TSA showed just a minor effect on the potency induced by cisplatin, but this modest effect may just be due to the low concentration of TSA used in the assay. On the other hand, we detected a significant enhancement in the potency of topotecan when co-incubated with SAHA or TSA in the cervix carcinoma cell line SiSo. These results are consistent with previous studies with other cell lines [51,52]. Even more interestingly, we discovered a highly significant potentiation of the antiproliferative potency of cisplatin and lomustine when SiSo cells were co-treated with **1a**, **3a**, and **5a**. This is the first report of a positive effect of HDAC inhibition on the toxicity of lomustine. Furthermore, all tested sirtuin inhibitors (except **5b**) increased the potency of topotecan. The effect to potentiate standard anticancer drugs can be related to the loosening of the chromatin structure. Due to the “open” chromatin structure, the accessibility of the DNA as the drug target may be promoted and cytotoxicity of DNA-damaging chemotherapeutics such as cisplatin, lomustine, and topotecan can be increased [53,54]. However, further studies are required to confirm this hypothesis. In the future, it would even be worthwhile to investigate whether the HDAC inhibitors possess a similar modulating potential when combined with anticancer drugs in more cancer cell lines.

## 5. Conclusions

In summary, insights have been gained into the connection between HDAC/Sirt expression and anticancer drug sensitivity. These studies provide possible clues for the optimisation of the anticancer drug therapy. With the current work, it has been shown that the expression of several HDAC/Sirt isoenzymes correlates with the sensitivity towards a number of anticancer drugs, such as platinum compounds, topoisomerase inhibitors, or other cytostatic agents of natural origin. In most cases, a positive correlation to the relative protein expression with the GI_50_ values of the drug was detected, indicating that cancer cells with low enzyme expression are especially sensitive to anticancer drug treatment. Furthermore, we demonstrated that the inhibition of Sirt2 significantly increased the potency of cisplatin, lomustine, and topotecan in vitro. Based on this observation, a set of recently discovered Sirt2 inhibitors was used to gain first insights into their potential benefit in combination with such anticancer drugs.

## Figures and Tables

**Table 1:** GI_50_ values for the inhibition of proliferation in 16 human cancer cell lines caused by SAHA and TSA [mean (upper values) ± SD (lower values) of n>3 independent determinations] and their relative GI_50_ values (relative to the mean GI_50_ over all cell lines).

Cell Line	SAHA		TSA			SAHA		TSA	
	GI_50_ (nm)	rel. GI_50_	GI_50_ (nm)	rel. GI_50_	Cell Line	GI_50_ (nm)	rel. GI_50_	GI_50_ (nm)	rel. GI_50_
MCF-7	527.8	0.64	33.55	0.45	BHY	598.7	0.73	52.36	0.71
	79.4		14.41			136.3		7.20	
MT-3	689.6	0.84	77.74	1.05	DanG	586.1	0.72	71.90	0.97
	114.9		14.48			35.7		11.33	
SiSo	909.3	1.11	60.13	0.81	Pa-Tu-8902	1536.6	1.88	123.92	1.67
	126.6		3.56			248.5		9.36	
Kyse-70	602.0	0.74	50.56	0.68	YAPC	1210.4	1.48	72.83	0.98
	67.5		7.69			228.9		38.57	
Kyse-510	1862.6	2.28	174.83	2.36	5637	667.2	0.82	78.71	1.06
	210.3		40.01			239.4		38.98	
Kyse-520	501.5	0.61	78.14	1.06	RT-4	873.6	1.07	98.75	1.33
	45.4		29.97			115.5		22.38	
A427	228.3	0.28	24.89	0.34	RT-112	825.9	1.01	92.68	1.25
	158.0		24.26			106.6		16.12	
LCLC-103H	511.8	0.63	38.72	0.52	A2780	964.9	1.18	54.47	0.74
	74.2		13.13			165.0		6.98	

**Table 2:** GI_50_ values for the antiproliferative activity of six anticancer drugs in 11 human cancer cell lines [mean (upper values) ± SD (lower values, gray) of n=3].

	MCF-7	MT-3	Kyse-510	Kyse-520	A427	LCLC-103H	BHY	DanG	Pa-Tu-8902	YAPC	5637
Imatinib (μM)	11.61	11.49	13.38	9.24	6.47	9.29	13.20	6.78	16.56	11.77	12.98
	3.75	0.27	1.16	1.30	5.63	3.97	0.91	1.21	0.71	1.14	1.66
Lomustine (μM)	55.83	3.51	23.30	16.03	11.47	22.29	41.00	39.40	37.45	46.04	15.14
	8.63	0.35	1.16	4.91	3.06	6.24	4.65	10.80	1.97	5.86	3.75
Temozolomide (μM)	>400	>400	22.82	>400	6.07	71.27	>400	>400	211.92	>400	>400
			2.23		0.52	7.40			35.32		
Bortezomib (nM)	4.51	6.16	9.00	8.69	7.79	5.69	4.89	6.24	4.10	6.34	8.65
	2.03	1.46	4.21	3.39	0.78	1.37	1.58	0.51	0.28	1.09	1.22
Paclitaxel (nM)	1.22	3.61	1.68	1.19	1.42	1.17	1.24	1.01	1.46	1.63	1.23
	0.33	0.70	0.31	0.18	0.36	0.04	0.11	0.26	0.17	0.08	0.04
Topotecan (nM)	18.71	10.24	56.30	11.70	21.98	15.41	21.02	52.95	12.12	20.67	13.04
	2.82	4.15	1.36	1.66	7.20	0.88	1.33	2.08	0.98	1.12	0.81

**Table 3:** GI_50_ and relative GI_50_ values (CGI) for cisplatin, lomustine, and topotecan in combination with HDAC inhibitors (SAHA: 0.3 μM, TSA: 0.03μM, **2a**: 5 μM, **1a** and **2a**–**5c**: 50 μM) in SiSo cells [mean ± SD of n>3]. Statistical significant values are shown in boldface while background colors were applied for easier visualization.

	Cisplatin		Lomustine		Topotecan	
	GI_50_ (μM)	CGI	GI_50_ (μM)	CGI	GI_50_ (μM)	CGI
W/O	237.9	1.000	4.93	1.000	20.15	1.000
	60.8		0.61		2.03	
SAHA	156.3	0.805	4.90	0.948	16.31	**0.634**
	18.2	0.070	0.36	0.023	1.26	0.318
TSA	177.5	0.914	5.15	0.995	16.52	**0.804**
	25.6	0.103	0.50	0.045	0.94	0.032
**1a**	134.9	**0.428**	2.40	**0.495**	13.54	**0.700**
	50.9	0.072	0.28	0.074	0.58	0.050
**2a**	184.2	0.875	4.10	0.853	10.03	**0.518**
	13.7	0.066	0.99	0.069	0.59	0.029
**3a**	189.7	**0.606**	2.31	**0.473**	11.20	**0.578**
	70.8	0.112	0.40	0.055	0.80	0.029
**4a**	265.3	0.855	4.05	0.828	14.64	**0.754**
	64.4	0.053	0.70	0.092	1.44	0.041
**5a**	120.6	**0.566**	2.85	**0.591**	12.83	**0.659**
	31.8	0.089	0.72	0.042	1.68	0.035
**5b**	308.0	1.002	5.18	1.068	18.84	0.967
	47.5	0.025	0.76	0.164	2.68	0.039
**5c**	275.3	0.890	4.56	0.929	15.44	**0.790**
	55.0	0.048	0.72	0.039	3.12	0.074

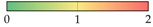

## Data Availability

All primary data are available at Supplementary Materials.

## Supplementary Materials

The following are available at https://www.mdpi.com/article/10.3390/cancers14010187/s1, Figure S1: Representative electropherogram showing the total protein content of the panel of 17 cancer cell lines used as internal controls in all Western blotting, as developed according to the TGX Stain Free Gels systems from Bio-Rad; Figure S2: Western blots for the panel of 17 cancer cell lines for HDAC1, marked with antibody #5356 from Cell Signaling Technology; Figure S3: Western blots for the panel of 17 cancer cell lines for HDAC2, marked with antibody #5113 from Cell Signaling Technology; Figure S4: Western blots for the panel of 17 cancer cell lines for HDAC4, marked with antibody #7628 from Cell Signaling Technology; Figure S5: Western blots for the panel of 17 cancer cell lines for HDAC6, marked with antibody #7558 from Cell Signaling Technology; Figure S6: Western blots for the panel of 17 cancer cell lines for Sirt1, marked with antibody #9475 from Cell Signaling Technology; Figure S7: Western blots for the panel of 17 cancer cell lines for Sirt2, marked with antibody #12650 from Cell Signaling Technology; Figure S8: Western blots for the panel of 17 cancer cell lines for Sirt3, marked with antibody #5490 from Cell Signaling Technology; Figure S9: Western blots for the panel of 17 cancer cell lines for Sirt5, marked with antibody #8782 from Cell Signaling Technology; Figure S10: Western blots for the panel of 17 cancer cell lines for Sirt6, marked with antibody #12486 from Cell Signaling Technology; Figure S11: Western blots for the panel of 17 cancer cell lines for Sirt7, marked with antibody #5360 from Cell Signaling Technology; Figure S12: Univariate correlation matrix of the Spearman correlation coefficients for the expression of HDAC/Sirt isoenzyme proteins; Figure S13: Univariate correlation matrix of the Spearman correlation coefficients with the NCI data for the expression of HDAC/Sirt isoenzyme mRNA; Figure S14: Univariate correlation matrix of the Spearman correlation coefficients for the expression of the HDAC/Sirt isoenzyme protein with anticancer drug potency expressed as GI_50_ values and the doubling time of cancer cells; Figure S15: Univariate correlation matrix of the Spearman correlation coefficients correlation coefficients with the NCI data for the expression of the HDAC/Sirt isoenzyme mRNA with anticancer drug potency expressed as GI_50_; Figure S16: Univariate correlation matrix of the Pearson with FDR correction correlation for the expression of HDAC/Sirt isoenzyme proteins; Figure S17: Univariate correlation matrix of the Pearson with FDR correction correlation with the NCI data for the expression of HDAC/Sirt isoenzyme mRNA; Figure S18: Univariate correlation matrix of the Pearson with FDR correction correlation for the expression of the HDAC/Sirt isoenzyme protein with anticancer drug potency expressed as GI_50_ values and the doubling time of cancer cells; Figure S19: Univariate correlation matrix of the Pearson with FDR correction correlation for the expression of the HDAC/Sirt isoenzyme protein with anticancer drug potency expressed as GI_50_; Table S1: Relative HDAC/Sirt isoenzyme protein expression of various cancer cell lines; Table S2: *R* and *p*-values of Pearson correlation matrix for HDAC isoenzyme protein expression; Table S3: *R* and *p*-values of Spearman correlation matrix for HDAC isoenzyme protein expression; Table S4: *R* and *p*-values of Pearson correlation matrix for HDAC isoenzyme mRNA expression with data of the NCI 60 cancer cell line program; Table S5: Doubling times in hours of cancer cell lines; Table S6. *R* and *p*-values of univariate correlation of HDAC isoenzyme protein expression with anticancer drug potency expressed as GI_50_ values and the doubling time of cancer cells; Table S7: *R* and *p*-values of Pearson correlation of HDAC isoenzyme mRNA expression with data of the NCI 60 cancer cell line program with anticancer drug potency expressed as GI_50_ values; Table S8: Localization of HDAC isoenzymes across the chromosomes and corresponding reference sequence; from on UCSC Genome Browser.

## Abbreviations

The following abbreviations are used in this manuscript:

BSA	bovine serum albumin
CGI	relative combination growth inhibition at 50%
FDR	false discovery rate
GI_50_	growth inhibition at 50%
HAT	histone acetyltransferase
HDAC	histone deacetylase
NCI	National Cancer Institute
OLS	ordinary least squares
SAHA	suberanilohydroxamic acid (vorinostat)
SD	standard deviation
Sirt	sirtuin
TBST	Tris buffered saline/tween buffer
TSA	trichostatin A
