# Supplementary material for: Correlation Analysis of Protein Expression of 10 HDAC/Sirtuin Isoenzymes with Sensitivities of 23 Anticancer Drugs in 17 Cancer Cell Lines and Potentiation of Drug Activity by Co-Treatment with HDAC Inhibitors
Source: Cancers (Basel). 2021 Dec 31;14(1):187. doi: 10.3390/cancers14010187 (PMC8750037; doi:10.3390/cancers14010187)

Supplementary Materials

Correlation Analysis of Protein Expression of 10 HDAC/Sirtuin Isoenzymes with Sensitivities of 23 Anticancer Drugs in 17 Cancer Cell Lines and Potentiation of Drug Activity by Co-Treatment with HDAC Inhibitors

Steven Behnisch-Cornwell †, Christoph W. Grathwol, Lukas Schulig, Anika Voigt, Daniel Baecker, Andreas Link and Patrick J. Bednarski *

Department of Pharmaceutical/Medicinal Chemistry, Institute of Pharmacy, University of Greifswald, Friedrich-Ludwig-Jahn-Straße 17, 17489 Greifswald, Germany; steven.behnisch@web.de (S.B.-C.); christoph.grathwol@kit.edu (C.W.G.); lukas.schulig@uni-greifswald.de (L.S.); anika.voigt@uni-greifswald.de (A.V.); daniel.baecker@uni-greifswald.de (D.B.); link@uni-greifswald.de (A.L.)

* Correspondence: bednarsk@uni-greifswald.de; Tel.: +49-3834-86-4883

† Present address: AstraZeneca GmbH, Tinsdaler Weg 183, 22880 Wedel, Germany.

Table S1. Relative HDAC/Sirt isoenzyme protein expression of various cancer cell lines.

Table S2. R and p-values of Pearson correlation matrix for HDAC isoenzyme protein expression.

Table S3. R and p-values of Spearman correlation matrix for HDAC isoenzyme protein expression.

Table S4. R and p-values of Pearson correlation matrix for HDAC isoenzyme mRNA expression with data of the NCI 60 cancer cell line program.

Table S5. Doubling times in hours of cancer cell lines.

Table S6. R and p-values of univariate correlation of HDAC isoenzyme protein expression with anticancer drug potency expressed as GI_{50} values and the doubling time of cancer cells.

Table S7. R and p-values of Pearson correlation of HDAC isoenzyme mRNA expression with data of the NCI 60 cancer cell line program with anticancer drug potency expressed as GI_{50} values.

Table S8. Localization of HDAC isoenzymes across the chromosomes and corresponding reference sequence; from on UCSC Genome Browser.

Figure S1. Representative electropherogram showing the total protein content of the panel of 17 cancer cell lines used as internal controls in all Western blotting, as developed according to the TGX Stain Free Gels systems from Bio-Rad.

Figure S2. Western blots for the panel of 17 cancer cell lines for HDAC1, marked with antibody #5356 from Cell Signaling Technology.

Figure S3. Western blots for the panel of 17 cancer cell lines for HDAC2, marked with antibody #5113 from Cell Signaling Technology.

Figure S4. Western blots for the panel of 17 cancer cell lines for HDAC4, marked with antibody #7628 from Cell Signaling Technology.

Figure S5. Western blots for the panel of 17 cancer cell lines for HDAC6, marked with antibody #7558 from Cell Signaling Technology.

Figure S6. Western blots for the panel of 17 cancer cell lines for Sirt1, marked with antibody #9475 from Cell Signaling Technology.

Figure S7. Western blots for the panel of 17 cancer cell lines for Sirt2, marked with antibody #12650 from Cell Signaling Technology.

Figure S8. Western blots for the panel of 17 cancer cell lines for Sirt3, marked with antibody #5490 from Cell Signaling Technology.

Figure S9. Western blots for the panel of 17 cancer cell lines for Sirt5, marked with antibody #8782 from Cell Signaling Technology.

Figure S10. Western blots for the panel of 17 cancer cell lines for Sirt6, marked with antibody #12486 from Cell Signaling Technology.

Figure S11. Western blots for the panel of 17 cancer cell lines for Sirt7, marked with antibody #5360 from Cell Signaling Technology.

Figure S12.Univariate correlation matrix of the Spearman correlation coefficients for the expression of HDAC/Sirt isoenzyme proteins.

Figure S13. Univariate correlation matrix of the Spearman correlation coefficients with the NCI data for the expression of HDAC/Sirt isoenzyme mRNA.

Figure S14. Univariate correlation matrix of the Spearman correlation coefficients for the expression of the HDAC/Sirt isoenzyme protein with anticancer drug potency expressed as GI_{50} values and the doubling time of cancer cells.

Figure S15. Univariate correlation matrix of the Spearman correlation coefficients with the NCI data for the expression of the HDAC/Sirt isoenzyme mRNA with anticancer drug potency expressed as GI_{50}.

Figure S16. Univariate correlation matrix of the Pearson with FDR correction correlation for the expression of HDAC/Sirt isoenzyme proteins.

Figure S17. Univariate correlation matrix of the Pearson with FDR correction correlation with the NCI data for the expression of HDAC/Sirt isoenzyme mRNA.

Figure S18. Univariate correlation matrix of the Pearson with FDR correction correlation for the expression of the HDAC/Sirt isoenzyme protein with anticancer drug potency expressed as GI_{50} values and the doubling time of cancer cells.

Figure S19. Univariate correlation matrix of the Pearson with FDR correction correlation for the expression of the HDAC/Sirt isoenzyme protein with anticancer drug potency expressed as GI_{50}.

Table S1. Relative HDAC/Sirt isoenzyme protein expression of various cancer cell lines (relative to the mean expression over all cell lines) of n ≥ 3 independent determinations.

HDAC1	EFM-19	MCF-7	MT-3	SiSo	Kyse-70	Kyse-510	Kyse-520	A427	EPLC-272H
19_1_19	0.7098	1.4577	1.1490	0.6941	1.1442	1.6578	1.0719	2.2210	1.2739
19_1_26	1.1100	1.0000	1.0600	0.5000	0.8700	0.6500	1.0100	0.7700	1.5200
19_1_30	0.4700	2.1000	0.6900	0.4400	1.1200	1.2000	1.2000	2.8000	1.2600
19_1_32	0.5170	1.7850	1.4300	0.6460	0.9490	1.1800	1.0940	2.1200	1.3600
19_1_45	0.5980	2.2470	1.6030	0.6380	0.9710	1.2930	0.8380	(outlier)	1.0070
Mean	0.6810	1.7180	1.1860	0.5836	1.0110	1.1960	1.0430	1.9780	1.2840
Std. Deviation	0.2565	0.5034	0.3524	0.1080	0.1172	0.3609	0.1334	0.8591	0.1863
**HDAC1**	LCLC-103H	BHY	DanG	Pa-Tu-8902	YAPC	5637	RT-4	RT-112	
19_1_19	1.0092	1.6329	1.0189	(outlier)	0.9998	(outlier)	0.3375	0.4917	
19_1_26	1.1400	2.2700	1.3400	1.0400	1.6100	0.3600	0.2700	0.4700	
19_1_30	1.0300	1.6100	0.6900	0.3300	(outlier)	1.0200	0.4100	0.4400	
19_1_32	0.6740	1.5270	0.9500	0.3860	0.9710	0.4010	0.2610	0.7500	
19_1_45	1.1950	1.8620	1.1840	0.9350	1.2010	0.3620	0.2630	0.6460	
Mean	1.0100	1.7800	1.0370	0.6728	1.1950	0.5358	0.3083	0.5595	
Std. Deviation	0.2027	0.3006	0.2458	0.3667	0.2947	0.3234	0.0651	0.1329	
**HDAC2**	EFM-19	MCF-7	MT-3	SiSo	Kyse-70	Kyse-510	Kyse-520	A427	EPLC-272H
19_1_26	1.2100	0.7100	1.5800	0.8500	1.1000	1.9600	0.8300	1.0000	0.9700
19_1_30	0.8500	1.0600	0.7900	1.0400	1.0900	2.4500	0.5700	0.4300	0.3100
19_1_32	1.6010	1.3650	1.4670	1.0880	0.6870	1.3620	0.7240	1.2060	0.7640
19_1_45	1.8990	1.1250	1.0420	1.2180	0.8300	1.2990	0.8610	(outlier)	0.8820
19_1_58	1.6210	(outlier)	1.7610	0.8770	0.8800	1.7030	0.9370	0.6500	(outlier)
Mean	1.4360	1.0650	1.3280	1.0150	0.9174	1.7550	0.7844	0.8215	0.7315
Std. Deviation	0.4094	0.2706	0.4005	0.1528	0.1770	0.4717	0.1422	0.3476	0.2934
**HDAC2**	LCLC-103H	BHY	DanG	Pa-Tu-8902	YAPC	5637	RT-4	RT-112	

19_1_26	1.2700	1.0200	1.4300	1.1600	0.4200	0.9700	0.0900	0.4400	
19_1_30	1.5300	0.9300	1.3200	0.9700	(outlier)	0.5600	0.4500	0.5400	
19_1_32	0.6030	0.8840	1.6430	1.0190	0.6010	1.4880	0.3040	0.1940	
19_1_45	1.0620	0.7310	1.0830	0.9310	0.6080	1.9330	0.3870	0.4960	
19_1_58	0.8410	0.7690	1.3940	0.9930	0.5450	1.3500	0.4590	0.3500	
Mean	1.0610	0.8668	1.3740	1.0150	0.5435	1.2600	0.3380	0.4040	
Std. Deviation	0.3612	0.1181	0.2022	0.0875	0.0870	0.5213	0.1519	0.1372	
**HDAC4**	EFM-19	MCF-7	MT-3	SiSo	Kyse-70	Kyse-510	Kyse-520	A427	EPLC-272H
19_1_15	1.4710	0.4620	0.5550	0.9530	0.8600	1.2820	0.7190	1.9270	0.2920
19_1_19	1.3769	0.3783	0.8203	1.0207	1.6994	2.0632	1.1484	1.5772	0.4595
19_1_26	1.2500	0.1100	1.2400	0.6800	2.1100	1.9500	0.9700	1.6100	0.8300
19_1_30	1.0494	0.5055	0.3900	0.6197	2.0948	1.9577	1.0241	1.4057	0.4340
Mean	1.2870	0.3639	0.7513	0.8184	1.6910	1.8130	0.9654	1.6300	0.5039
Std. Deviation	0.1824	0.1773	0.3709	0.1981	0.5857	0.3579	0.1804	0.2173	0.2296
**HDAC4**	LCLC-103H	BHY	DanG	Pa-Tu-8902	YAPC	5637	RT-4	RT-112	
19_1_15	0.7680	0.2140	1.7750	1.0090	1.6740	1.0580	0.7760	1.2070	
19_1_19	0.4242	0.3090	1.4307	(outlier)	2.3726	(outlier)	0.5775	0.9938	
19_1_26	0.7400	0.3300	1.4300	0.9000	1.7500	0.1800	0.3500	0.5700	
19_1_30	0.5455	0.2474	1.5795	0.7668	(outlier)	1.9391	0.7906	1.3559	
Mean	0.6194	0.2751	1.5540	0.8919	1.9320	1.0590	0.6235	1.0320	
Std. Deviation	0.1635	0.0538	0.1634	0.1213	0.3833	0.8796	0.2066	0.3418	
**HDAC6**	EFM-19	MCF-7	MT-3	SiSo	Kyse-70	Kyse-510	Kyse-520	A427	EPLC-272H
19_1_26	1.0700	0.1100	1.1500	1.1100	1.0000	1.0300	0.3900	2.7900	1.2000
19_1_30	1.5600	0.8300	0.2300	1.1700	0.6200	0.3500	0.0300	3.6400	0.1300
19_1_32	0.8500	0.7350	1.7720	1.4050	0.8340	1.0010	0.2120	2.5380	0.4930
19_1_45	1.7940	0.6040	1.6500	1.6880	0.7980	1.1120	0.3040	(outlier)	0.3640
19_1_58	1.1200	(outlier)	0.4380	3.7970	0.5010	0.3520	0.1960	4.4810	(outlier)
19_1_72	1.2900	0.1700	0.3000	3.9800	0.4600	0.7600	0.0100	3.8200	0.1200

Mean	1.2810	0.4898	0.9233	2.1920	0.7022	0.7675	0.1903	3.4540	0.4614
Std. Deviation	0.3456	0.3299	0.6935	1.3310	0.2103	0.3433	0.1493	0.7911	0.4423
**HDAC6**	LCLC-103H	BHY	DanG	Pa-Tu-8902	YAPC	5637	RT-4	RT-112	
19_1_26	1.2300	0.8900	0.2700	0.6600	1.9600	0.4200	0.5800	1.1600	
19_1_30	0.6200	0.3400	0.1100	0.3900	(outlier)	4.1500	0.8600	1.8900	
19_1_32	1.0080	0.9380	0.2440	0.1060	3.1210	0.1720	0.7230	0.8460	
19_1_45	1.0700	1.0460	0.1340	0.6580	1.5870	1.5620	0.9190	1.5370	
19_1_58	1.0560	0.6370	0.1730	0.4740	1.9300	0.5390	0.2080	0.7560	
19_1_72	0.3000	0.5600	0.0100	(outlier)	4.1500	(outlier)	0.0000	1.0600	
Mean	0.8807	0.7352	0.1568	0.4576	2.5500	1.3690	0.5483	1.2080	
Std. Deviation	0.3493	0.2677	0.0948	0.2289	1.0660	1.6430	0.3695	0.4320	
**Sirt1**	EFM-19	MCF-7	MT-3	SiSo	Kyse-70	Kyse-510	Kyse-520	A427	EPLC-272H
19_1_15	0.4120	1.6240	0.9320	1.6920	1.2130	0.6530	0.5790	4.5220	0.3390
19_1_19	0.3902	0.7098	1.1877	1.1766	1.0739	0.6264	0.6444	5.8579	1.2907
19_1_26	0.4200	0.4600	1.5700	1.1300	1.5500	1.0100	0.7600	4.2300	1.2900
19_1_30	0.4400	1.4100	0.5200	0.9000	1.4400	1.0100	0.9100	3.9200	1.1800
19_1_32	0.3180	0.8890	1.0630	1.0220	1.3400	0.9460	1.0210	2.6500	1.4330
19_1_45	0.4490	1.0030	1.3830	1.2130	1.1640	0.7020	0.7960	(outlier)	1.3010
Mean	0.4049	1.0160	1.1090	1.1890	1.2970	0.8246	0.7851	4.2360	1.1390
Std. Deviation	0.0474	0.4345	0.3672	0.2715	0.1793	0.1829	0.1639	1.1540	0.4000
**Sirt1**	LCLC-103H	BHY	DanG	Pa-Tu-8902	YAPC	5637	RT-4	RT-112	
19_1_15	1.1230	0.6940	0.4240	1.5420	0.5470	0.4480	0.1520	0.1070	
19_1_19	1.0535	0.8406	0.3642	(outlier)	0.9501	(outlier)	0.2156	0.2864	
19_1_26	1.0200	0.6900	0.3500	1.0600	0.6800	0.4100	0.1400	0.2400	
19_1_30	1.0900	0.9500	0.4700	1.1000	(outlier)	0.8400	0.0100	0.3000	
19_1_32	1.2450	1.2300	0.6210	0.7600	1.1210	0.5170	0.3880	0.4360	
19_1_45	1.4160	1.0050	0.7110	1.4170	1.1540	1.0510	0.4110	0.3920	
Mean	1.1580	0.9016	0.4900	1.1760	0.8904	0.6532	0.2194	0.2936	
Std. Deviation	0.1483	0.2061	0.1457	0.3100	0.2685	0.2797	0.1548	0.1164	

**Sirt2**	EFM-19	MCF-7	MT-3	SiSo	Kyse-70	Kyse-510	Kyse-520	A427	EPLC-272H
19_1_15	2.2040	1.5450	0.7370	1.1980	0.4020	0.3710	0.4310	0.5970	0.5390
19_1_19	2.3136	1.3385	0.7097	1.1276	0.7356	0.5985	0.6322	0.6294	0.6601
19_1_26	1.9500	1.2800	0.5800	1.0000	0.5600	0.2200	0.4100	0.6700	0.8100
19_1_30	1.6800	1.3100	0.7400	1.0000	0.5600	0.2200	0.6200	0.6200	0.6300
Mean	2.0370	1.3680	0.6917	1.0810	0.5644	0.3524	0.5233	0.6291	0.6598
Std. Deviation	0.2825	0.1202	0.0757	0.0983	0.1363	0.1789	0.1191	0.0305	0.1126
**Sirt2**	LCLC-103H	BHY	DanG	Pa-Tu-8902	YAPC	5637	RT-4	RT-112	
19_1_15	0.8520	0.6610	1.0370	0.2600	1.2110	1.3990	2.2160	1.3400	
19_1_19	0.4892	0.8161	1.0412	(outlier)	1.5604	(outlier)	2.0139	1.5539	
19_1_26	0.8000	0.9100	0.8800	0.3200	1.2100	1.1600	2.8300	1.4300	
19_1_30	0.7600	0.6800	1.0800	0.2500	(outlier)	1.4200	2.8300	1.4600	
Mean	0.7253	0.7668	1.0100	0.2767	1.3270	1.3260	2.4720	1.4460	
Std. Deviation	0.1618	0.1178	0.0885	0.0379	0.2020	0.1444	0.4210	0.0882	
**Sirt3**	EFM-19	MCF-7	MT-3	SiSo	Kyse-70	Kyse-510	Kyse-520	A427	EPLC-272H
19_1_15	0.4510	1.2340	2.1540	0.7810	1.7850	1.0310	0.6790	2.7330	0.5960
19_1_19	0.3494	1.2609	2.3490	0.8142	2.3233	1.1651	0.4160	3.6066	0.4640
19_1_26	0.1900	0.8300	2.6700	0.4400	2.3000	0.8100	0.5100	4.0000	0.8900
19_1_30	0.2800	1.4900	2.4500	0.4800	2.4000	0.6500	0.4800	3.6500	0.6300
Mean	0.3176	1.2040	2.4060	0.6288	2.2020	0.9140	0.5212	3.4970	0.6450
Std. Deviation	0.1103	0.2744	0.2148	0.1961	0.2813	0.2289	0.1123	0.5392	0.1783
**Sirt3**	LCLC-103H	BHY	DanG	Pa-Tu-8902	YAPC	5637	RT-4	RT-112	
19_1_15	1.1450	1.0630	0.8270	0.5850	0.5420	0.3500	0.6780	0.3670	
19_1_19	0.8486	1.2747	0.5198	(outlier)	0.8610	(outlier)	0.3953	0.2197	
19_1_26	0.9400	1.2000	0.6700	0.4800	0.5200	0.1300	0.3100	0.1100	
19_1_30	0.9700	0.8800	0.8800	0.4400	(outlier)	0.6100	0.4100	0.1500	
Mean	0.9759	1.1040	0.7242	0.5017	0.6410	0.3633	0.4483	0.2117	

Std. Deviation	0.1240	0.1734	0.1628	0.0749	0.1908	0.2403	0.1593	0.1130	
**Sirt5**	EFM-19	MCF-7	MT-3	SiSo	Kyse-70	Kyse-510	Kyse-520	A427	EPLC-272H
19_1_26	0.4200	0.8000	0.8300	0.3900	1.2900	1.3000	0.6400	1.1900	1.4000
19_1_30	0.3633	1.0639	1.4179	0.8495	1.4616	1.0524	0.5443	1.1173	0.8794
19_1_32	0.3380	0.8150	1.1070	0.5470	1.5360	1.8940	0.4940	0.8590	0.7400
19_1_45	0.4940	0.7060	0.9230	0.5960	1.0890	1.4650	0.9230	(outlier)	0.8200
19_1_58	0.3910	(outlier)	0.7220	0.2560	1.1170	1.9540	0.7260	0.7550	(outlier)
Mean	0.4013	0.8462	1.0000	0.5277	1.2990	1.5330	0.6655	0.9803	0.9598
Std. Deviation	0.0602	0.1529	0.2731	0.2244	0.1999	0.3865	0.1694	0.2067	0.2989
**Sirt5**	LCLC-103H	BHY	DanG	Pa-Tu-8902	YAPC	5637	RT-4	RT-112	
19_1_26	0.6000	1.3200	2.5700	1.5100	0.8500	0.2400	0.8000	0.8600	
19_1_30	0.1699	0.7653	3.9278	0.8666	(outlier)	1.2612	0.7343	0.3736	
19_1_32	0.5660	1.1120	3.0010	1.0620	1.1380	0.1000	0.6930	0.9990	
19_1_45	0.4840	0.9950	3.4530	1.6050	1.0550	0.2210	0.7560	0.8220	
19_1_58	0.3390	0.9430	3.0780	1.3600	1.0840	0.0600	0.7200	0.6180	
Mean	0.4318	1.0270	3.2060	1.2810	1.0320	0.3764	0.7407	0.7345	
Std. Deviation	0.1776	0.2059	0.5111	0.3096	0.1260	0.5005	0.0403	0.2435	
**Sirt6**	EFM-19	MCF-7	MT-3	SiSo	Kyse-70	Kyse-510	Kyse-520	A427	EPLC-272H
19_1_32	0.7760	1.1420	1.4870	0.7000	2.2410	0.7930	1.0940	2.1080	1.4580
19_1_45	0.4640	0.8140	1.2050	0.7320	2.3420	0.8330	0.8550	(outlier)	1.9230
19_1_58	0.8490	(outlier)	1.9420	0.5920	1.7530	0.7430	0.7240	1.9720	(outlier)
19_1_72	0.7200	0.7600	2.2200	0.6900	2.3000	0.5700	0.8300	3.1100	1.6100
19_1_82	0.8380	(outlier)	1.5191	(outlier)	1.6232	0.8631	0.8990	2.0122	1.0736
Mean	0.7294	0.9053	1.6750	0.6785	2.0520	0.7604	0.8804	2.3010	1.5160
Std. Deviation	0.1572	0.2067	0.4028	0.0604	0.3371	0.1156	0.1357	0.5426	0.3529
**Sirt6**	LCLC-103H	BHY	DanG	Pa-Tu-8902	YAPC	5637	RT-4	RT-112	
19_1_32	1.1520	0.6430	0.4390	0.2120	0.3900	1.0670	0.5740	0.7260	

19_1_45	1.2960	0.7280	0.5580	0.2850	0.4490	1.8270	0.6600	0.7480	
19_1_58	1.0760	0.6180	0.5230	0.2100	0.6340	2.4640	1.1580	0.6000	
19_1_72	1.3000	0.5800	0.3400	(outlier)	0.3600	(outlier)	0.7900	0.6800	
19_1_82	1.3071	0.7015	0.8264	0.5547	0.5203	1.5664	0.6416	0.8422	
Mean	1.2260	0.6541	0.5373	0.3154	0.4707	1.7310	0.7647	0.7192	
Std. Deviation	0.1060	0.0605	0.1822	0.1633	0.1100	0.5815	0.2333	0.0891	
**Sirt7**	EFM-19	MCF-7	MT-3	SiSo	Kyse-70	Kyse-510	Kyse-520	A427	EPLC-272H
19_1_30	0.6800	1.6800	0.4900	0.6700	0.3800	1.4100	1.2200	2.0900	0.8400
19_1_32	0.4450	1.5310	1.1300	0.9530	0.4550	1.2640	1.2890	1.8840	1.1710
19_1_45	0.5420	1.6400	1.1230	0.9550	0.4790	1.2480	1.1500	(outlier)	0.9690
19_1_58	0.8260	(outlier)	1.7810	1.0290	0.7030	0.8000	0.8850	1.3670	(outlier)
19_1_72	0.8900	1.6000	1.7400	1.1700	0.7200	1.0600	0.9900	1.8500	0.8600
19_1_82	0.7290	(outlier)	1.4020	(outlier)	0.5380	1.5260	0.9850	1.3330	0.7610
Mean	0.6853	1.6130	1.2780	0.9554	0.5458	1.2180	1.0870	1.7050	0.9202
Std. Deviation	0.1685	0.0635	0.4793	0.1823	0.1381	0.2586	0.1569	0.3369	0.1587
**Sirt7**	LCLC-103H	BHY	DanG	Pa-Tu-8902	YAPC	5637	RT-4	RT-112	
19_1_30	1.5700	1.2200	0.6800	1.5100	(outlier)	0.9100	0.7200	0.5300	
19_1_32	1.4070	1.2940	0.7820	0,651	1.0300	0.4980	0.5250	0.6890	
19_1_45	1.6870	1.0910	0.7600	1.6890	0,813	1.1410	0.5880	0.5840	
19_1_58	1.3310	1.1720	0.7750	1.5100	1.0840	1.2940	0.9960	0.5910	
19_1_72	1.3000	1.2200	0.7200	(outlier)	1.0400	(outlier)	0.7100	0.8500	
19_1_82	1.7020	1.1110	0.5560	1.8060	1.0210	0.6810	0.5580	0.7500	
Mean	1.5000	1.1850	0.7122	1.6290	1.0440	0.9048	0.6828	0.6657	
Std. Deviation	0.1777	0.0759	0.0856	0.1452	0.0279	0.3251	0.1730	0.1202	

Table S2. R and p-values of Pearson correlation matrix for HDAC isoenzyme protein expression.

	**HDAC1**		**HDAC2**		**HDAC4**		**HDAC6**	
**Isoenzyme**	R	p	R	p	R	p	R	p
**HDAC1**	1.000	0.000	0.209	0.473	0.032	0.913	0.226	0.438
**HDAC2**	0.209	0.473	1.000	0.000	0.166	0.570	-0.229	0.430
**HDAC4**	0.032	0.913	0.166	0.570	1.000	0.000	0.374	0.188
**HDAC6**	0.226	0.438	-0.229	0.430	0.374	0.188	1.000	0.000
**Sirt1**	0.627	0.016	0.024	0.936	0.265	0.361	0.708	0.005
**Sirt2**	-0.526	0.053	-0.588	0.027	-0.300	0.298	-0.032	0.915
**Sirt3**	0.643	0.013	0.155	0.596	0.206	0.479	0.410	0.146
**Sirt5**	0.181	0.536	0.389	0.169	0.455	0.102	-0.282	0.328
**Sirt6**	0.228	0.434	0.139	0.634	0.172	0.557	0.323	0.260
**Sirt7**	0.730	0.003	0.274	0.343	-0.236	0.417	0.303	0.292
	**Sirt1**		**Sirt2**		**Sirt3**		**Sirt5**	
**Isoenzyme**	R	p	R	p	R	p	R	p
**HDAC1**	0.627	0.016	-0.526	0.053	0.643	0.013	0.181	0.536
**HDAC2**	0.024	0.936	-0.588	0.027	0.155	0.596	0.389	0.169
**HDAC4**	0.265	0.361	-0.300	0.298	0.206	0.479	0.455	0.102
**HDAC6**	0.708	0.005	-0.032	0.915	0.410	0.146	-0.282	0.328
**Sirt1**	1.000	0.000	-0.404	0.152	0.840	0.000	-0.083	0.778
**Sirt2**	-0.404	0.152	1.000	0.000	-0.465	0.094	-0.186	0.525
**Sirt3**	0.840	0.000	-0.465	0.094	1.000	0.000	0.077	0.793
**Sirt5**	-0.083	0.778	-0.186	0.525	0.077	0.793	1.000	0.000
**Sirt6**	0.665	0.009	-0.333	0.245	0.774	0.001	-0.213	0.464
**Sirt7**	0.605	0.022	-0.371	0.192	0.482	0.081	-0.255	0.379
	**Sirt6**		**Sirt7**					
**Isoenzyme**	R	p	R	p				
**HDAC1**	0.228	0.434	0.730	0.003				
**HDAC2**	0.139	0.634	0.274	0.343				
**HDAC4**	0.172	0.557	-0.236	0.417				
**HDAC6**	0.323	0.260	0.303	0.292				
**Sirt1**	0.665	0.009	0.605	0.022				

**Sirt2**	-0.333	0.245	-0.371	0.192				
**Sirt3**	0.774	0.001	0.482	0.081				
**Sirt5**	-0.213	0.464	-0.255	0.379				
**Sirt6**	1.000	0.000	0.248	0.393				
**Sirt7**	0.248	0.393	1.000	0.000				

Table S3. R and p-values of Spearman correlation matrix for HDAC isoenzyme protein expression.

	HDAC1		HDAC2		HDAC4		HDAC6	
**Isoenzyme**	R	p	R	p	R	p	R	p
**HDAC1**	1.000	0.000	0.191	0.513	0.064	0.829	0.033	0.911
**HDAC2**	0.191	0.513	1.000	0.000	0.059	0.840	-0.165	0.573
**HDAC4**	0.064	0.829	0.059	0.840	1.000	0.000	0.314	0.274
**HDAC6**	0.033	0.911	-0.165	0.573	0.314	0.274	1.000	0.000
**Sirt1**	0.455	0.102	0.174	0.553	0.002	0.994	0.371	0.191
**Sirt2**	-0.446	0.110	-0.393	0.164	-0.301	0.296	0.090	0.759
**Sirt3**	0.719	0.004	0.341	0.233	-0.051	0.864	0.015	0.958
**Sirt5**	0.525	0.054	0.248	0.392	0.446	0.110	-0.266	0.358
**Sirt6**	-0.007	0.982	0.130	0.659	-0.015	0.958	0.125	0.670
**Sirt7**	0.710	0.004	0.301	0.296	-0.275	0.342	0.182	0.533
	**Sirt1**		**Sirt2**		**Sirt3**		**Sirt5**	
**Isoenzyme**	R	p	R	p	R	p	R	p
**HDAC1**	0.455	0.102	-0.446	0.110	0.719	0.004	0.525	0.054
**HDAC2**	0.174	0.553	-0.393	0.164	0.341	0.233	0.248	0.392
**HDAC4**	0.002	0.994	-0.301	0.296	-0.051	0.864	0.446	0.110
**HDAC6**	0.371	0.191	0.090	0.759	0.015	0.958	-0.266	0.358
**Sirt1**	1.000	0.000	-0.473	0.088	0.776	0.001	0.037	0.899
**Sirt2**	-0.473	0.088	1.000	0.000	-0.512	0.061	-0.301	0.296
**Sirt3**	0.776	0.001	-0.512	0.061	1.000	0.000	0.459	0.098
**Sirt5**	0.037	0.899	-0.301	0.296	0.459	0.098	1.000	0.000
**Sirt6**	0.459	0.098	-0.354	0.215	0.424	0.131	-0.297	0.303
**Sirt7**	0.495	0.072	-0.332	0.246	0.604	0.022	-0.059	0.840
	**Sirt6**		**Sirt7**					
**Isoenzyme**	R	p	R	p				
**HDAC1**	-0.007	0.982	0.710	0.004				
**HDAC2**	0.130	0.659	0.301	0.296				
**HDAC4**	-0.015	0.958	-0.275	0.342				
**HDAC6**	0.125	0.670	0.182	0.533				
**Sirt1**	0.459	0.098	0.495	0.072				

**Sirt2**	-0.354	0.215	-0.332	0.246				
**Sirt3**	0.424	0.131	0.604	0.022				
**Sirt5**	-0.297	0.303	-0.059	0.840				
**Sirt6**	1.000	0.000	0.266	0.358				
**Sirt7**	0.266	0.358	1.000	0.000				

Table S4. R and p-values of Pearson correlation matrix for HDAC isoenzyme mRNA expression with data of the NCI 60 cancer cell line program.

	**HDAC1**		**HDAC2**		**HDAC4**		**HDAC6**	
**Isoenzyme**	R	p	R	p	R	p	R	p
**HDAC1**	1.000	0.000	0.354	0.006	0.192	0.145	0.364	0.005
**HDAC2**	0.354	0.006	1.000	0.000	0.197	0.135	0.139	0.290
**HDAC4**	0.192	0.145	0.197	0.135	1.000	0.000	0.258	0.049
**HDAC6**	0.364	0.005	0.139	0.290	0.258	0.049	1.000	0.000
**Sirt1**	0.269	0.039	0.269	0.038	0.076	0.566	0.316	0.014
**Sirt2**	-0.165	0.211	-0.242	0.063	-0.079	0.552	-0.050	0.706
**Sirt3**	0.083	0.531	0.102	0.438	0.150	0.255	0.036	0.782
**Sirt5**	-0.138	0.296	-0.115	0.382	0.003	0.983	0.032	0.810
**Sirt6**	0.182	0.168	-0.078	0.552	-0.061	0.645	-0.033	0.802
**Sirt7**	0.069	0.605	-0.043	0.743	0.037	0.783	0.028	0.830
	**Sirt1**		**Sirt2**		**Sirt3**		**Sirt5**	
**Isoenzyme**	R	p	R	p	R	p	R	p
**HDAC1**	0.269	0.039	-0.165	0.211	0.083	0.531	-0.138	0.296
**HDAC2**	0.269	0.038	-0.242	0.063	0.102	0.438	-0.115	0.382
**HDAC4**	0.076	0.566	-0.079	0.552	0.150	0.255	0.003	0.983
**HDAC6**	0.316	0.014	-0.050	0.706	0.036	0.782	0.032	0.810
**Sirt1**	1.000	0.000	-0.016	0.905	0.197	0.132	0.055	0.675
**Sirt2**	-0.016	0.905	1.000	0.000	0.080	0.541	0.252	0.053
**Sirt3**	0.197	0.132	0.080	0.541	1.000	0.000	-0.024	0.854
**Sirt5**	0.055	0.675	0.252	0.053	-0.024	0.854	1.000	0.000
**Sirt6**	-0.001	0.992	0.005	0.971	-0.103	0.434	0.174	0.184
**Sirt7**	0.079	0.551	-0.025	0.851	0.142	0.278	-0.057	0.666
	**Sirt6**		**Sirt7**					
**Isoenzyme**	R	p	R	p				
**HDAC1**	0.182	0.168	0.069					
**HDAC2**	-0.078	0.552	-0.043					
**HDAC4**	-0.061	0.645	0.037					
**HDAC6**	-0.033	0.802	0.028					
**Sirt1**	-0.001	0.992	0.079					

**Sirt2**	0.005	0.971	-0.025					
**Sirt3**	-0.103	0.434	0.142					
**Sirt5**	0.174	0.184	-0.057					
**Sirt6**	1.000	0.000	0.089					
**Sirt7**	0.089	0.497	1.000					

Table S5. Doubling times in hours of cancer cell lines [mean ± SD of n=5 independent determinations].

	doubling time [h]
**EFM-19**	90.1 ± 66.1
**MCF-7**	50.5 ± 6.7
**MT-3**	44.8 ± 14.3
**SiSo**	24.2 ± 0.9
**Kyse-70**	26.4 ± 1.5
**Kyse-510**	21.8 ± 3.2
**Kyse-520**	29.8 ± 5.8
**A427**	28.0 ± 1.9
**EPLC-272H**	100.9 ± 13.8
**LCLC-103H**	22.1 ± 2.2
**BHY**	28.6 ± 2.7
**DanG**	29.7 ± 3.0
**Pa-Tu-8902**	22.3 ± 1.6
**YAPC**	42.9 ± 3.0
**5637**	21.5 ± 1.2
**RT-4**	45.4 ± 7.0
**RT-112**	26.8 ± 2.9

Table S6. R and p-values of univariate correlation of HDAC isoenzyme protein expression with anticancer drug potency expressed as GI_{50} values and the doubling time of cancer cells.

	**Azacitidine**		**Bortezomib**		**Busulfan**		**Camptothecin**	
**Isoenzyme**	R	p	R	p	R	p	R	p
**HDAC1**	0.141	0.661	-0.524	0.183	-0.455	0.137	-0.025	0.939
**HDAC2**	-0.078	0.809	0.240	0.567	-0.486	0.109	0.377	0.227
**HDAC4**	-0.431	0.161	0.704	0.051	-0.400	0.198	0.311	0.325
**HDAC6**	-0.179	0.577	0.246	0.557	-0.287	0.367	-0.438	0.154
**Sirt1**	-0.342	0.277	0.116	0.785	-0.299	0.345	-0.272	0.393
**Sirt2**	0.337	0.284	-0.444	0.270	0.683	0.014	-0.287	0.367
**Sirt3**	-0.315	0.319	-0.129	0.760	-0.256	0.422	-0.262	0.411
**Sirt5**	-0.229	0.473	-0.173	0.682	-0.203	0.526	0.466	0.127
**Sirt6**	-0.475	0.118	0.293	0.481	-0.205	0.523	-0.426	0.168
**Sirt7**	0.246	0.441	-0.226	0.590	-0.228	0.475	-0.092	0.775

	**Carboplatin**		**Chlorambucil**		**Cisplatin**		**Colchicine**	
**Isoenzyme**	R	p	R	p	R	p	R	p
**HDAC1**	0.317	0.315	-0.132	0.683	0.273	0.391	0.087	0.789
**HDAC2**	-0.601	0.039	-0.175	0.587	-0.543	0.068	-0.136	0.673
**HDAC4**	0.177	0.583	0.249	0.436	0.180	0.577	-0.090	0.780
**HDAC6**	0.170	0.598	-0.560	0.058	0.204	0.525	-0.277	0.384
**Sirt1**	0.075	0.816	-0.419	0.175	0.092	0.777	-0.170	0.598
**Sirt2**	0.205	0.523	-0.080	0.805	0.058	0.857	0.028	0.930
**Sirt3**	0.103	0.750	-0.216	0.499	-0.012	0.969	0.083	0.797
**Sirt5**	-0.068	0.834	0.274	0.389	-0.134	0.677	0.049	0.881
**Sirt6**	-0.139	0.667	-0.327	0.299	-0.210	0.512	0.076	0.815
**Sirt7**	0.082	0.801	-0.430	0.163	0.146	0.651	-0.209	0.514

	**Doxorubicin**		**Etoposide**		**5-Fluorouracil**		**Hydroxyurea**	
**Isoenzyme**	R	p	R	p	R	p	R	p
**HDAC1**	-0.084	0.795	-0.455	0.137	-0.186	0.562	-0.082	0.799
**HDAC2**	-0.194	0.546	-0.469	0.124	-0.366	0.241	-0.515	0.086
**HDAC4**	0.196	0.542	-0.312	0.324	-0.438	0.155	0.022	0.947
**HDAC6**	-0.425	0.169	-0.204	0.524	-0.056	0.863	0.266	0.403
**Sirt1**	-0.298	0.347	-0.269	0.397	-0.234	0.464	0.066	0.839

**Sirt2**	-0.034	0.916	0.761	0.004	0.249	0.435	0.628	0.029
**Sirt3**	-0.030	0.927	-0.198	0.537	-0.465	0.128	0.047	0.884
**Sirt5**	0.034	0.916	-0.117	0.718	-0.554	0.062	-0.207	0.518
**Sirt6**	0.001	0.996	-0.169	0.599	-0.310	0.327	-0.058	0.859
**Sirt7**	-0.366	0.242	-0.304	0.336	0.191	0.552	-0.018	0.956

	**Imatinib**		**Lomustine**		**Melphalan**		**Methotrexate**	
**Isoenzyme**	R	p	R	p	R	p	R	p
**HDAC1**	-0.210	0.617	0.472	0.143	-0.110	0.735	0.067	0.837
**HDAC2**	0.344	0.404	0.115	0.737	-0.326	0.301	-0.137	0.672
**HDAC4**	-0.442	0.273	-0.362	0.274	0.173	0.591	-0.101	0.755
**HDAC6**	-0.327	0.428	-0.471	0.144	-0.327	0.300	-0.258	0.418
**Sirt1**	-0.526	0.180	-0.251	0.457	-0.090	0.780	-0.019	0.952
**Sirt2**	0.091	0.830	0.291	0.385	0.040	0.902	-0.314	0.321
**Sirt3**	-0.414	0.307	-0.256	0.448	0.186	0.562	-0.109	0.736
**Sirt5**	-0.467	0.243	0.365	0.270	0.003	0.992	-0.153	0.635
**Sirt6**	-0.226	0.591	-0.502	0.115	0.182	0.572	-0.061	0.849
**Sirt7**	-0.043	0.920	0.259	0.441	-0.362	0.247	0.070	0.828

	**Oxaliplatin**		**Podophyllotoxin**		**Taxol**		**Thiotepa**	
**Isoenzyme**	R	p	R	p	R	p	R	p
**HDAC1**	0.102	0.753	-0.064	0.844	-0.112	0.730	-0.433	0.159
**HDAC2**	0.421	0.172	-0.291	0.358	-0.217	0.497	-0.444	0.148
**HDAC4**	0.614	0.034	0.164	0.611	0.427	0.167	-0.376	0.229
**HDAC6**	-0.107	0.740	-0.077	0.811	-0.096	0.766	-0.398	0.200
**Sirt1**	-0.235	0.462	0.004	0.990	-0.044	0.892	-0.327	0.299
**Sirt2**	-0.185	0.565	-0.364	0.245	-0.281	0.376	0.561	0.058
**Sirt3**	-0.217	0.497	0.035	0.913	0.208	0.516	-0.211	0.511
**Sirt5**	0.814	0.001	-0.145	0.653	0.024	0.941	-0.210	0.513
**Sirt6**	-0.524	0.080	0.094	0.771	0.293	0.356	-0.133	0.681
**Sirt7**	-0.194	0.546	-0.313	0.322	-0.538	0.071	-0.248	0.437

	**Topotecan**		**Vinblastin**		**Doubling time**	
**Isoenzyme**	R	p	R	p	R	p
**HDAC1**	-0.049	0.909	-0.325	0.303	0.166	0.572

**HDAC2**	0.666	0.071	-0.173	0.591	-0.307	0.286
**HDAC4**	0.660	0.075	-0.199	0.536	-0.261	0.368
**HDAC6**	-0.194	0.644	-0.600	0.039	-0.107	0.716
**Sirt1**	-0.161	0.703	-0.340	0.280	-0.130	0.658
**Sirt2**	-0.259	0.536	-0.229	0.474	0.495	0.072
**Sirt3**	-0.206	0.624	-0.218	0.496	0.088	0.765
**Sirt5**	0.801	0.017	-0.154	0.633	0.000	0.999
**Sirt6**	-0.496	0.211	-0.012	0.970	-0.178	0.544
**Sirt7**	-0.313	0.450	-0.284	0.370	0.139	0.634

Table S7. R and p-vlaues of Pearson correlation of HDAC isoenzyme mRNA expression with data of the NCI 60 cancer cell line program with anticancer drug potency expressed as GI_{50} values.

	**Azacitidine**		**Bortezomib**		**Busulfan**		**Camptothecin**	
**Isoenzyme**	R	p	R	p	R	p	R	p
**HDAC1**	-0.064	0.632	-0.018	0.893	-0.030	0.824	-0.092	0.489
**HDAC2**	-0.035	0.793	0.104	0.433	-0.055	0.676	-0.115	0.380
**HDAC4**	0.046	0.728	-0.116	0.382	-0.197	0.135	-0.077	0.562
**HDAC6**	0.130	0.321	-0.015	0.911	-0.116	0.376	0.031	0.816
**Sirt1**	-0.070	0.595	-0.011	0.932	0.028	0.834	-0.084	0.521
**Sirt2**	-0.034	0.794	0.050	0.706	-0.204	0.118	-0.370	0.004
**Sirt3**	0.026	0.846	-0.123	0.353	-0.082	0.535	-0.032	0.806
**Sirt5**	0.161	0.219	0.010	0.942	-0.081	0.539	0.070	0.595
**Sirt6**	-0.027	0.837	-0.060	0.652	-0.121	0.356	0.141	0.284
**Sirt7**	-0.061	0.641	0.052	0.698	-0.003	0.983	-0.037	0.778

	**Carboplatin**		**Chlorambucil**		**Cisplatin**		**Colchicine**	
**Isoenzyme**	R	p	R	p	R	p	R	p
**HDAC1**	0.091	0.495	-0.076	0.567	0.023	0.864	-0.156	0.242
**HDAC2**	0.030	0.822	-0.096	0.466	-0.145	0.268	-0.286	0.028
**HDAC4**	0.045	0.736	-0.061	0.644	-0.021	0.876	-0.062	0.641
**HDAC6**	-0.006	0.961	-0.108	0.411	0.187	0.153	0.054	0.685
**Sirt1**	-0.045	0.736	-0.071	0.592	-0.046	0.725	-0.037	0.780
**Sirt2**	-0.179	0.170	-0.281	0.030	-0.092	0.483	0.123	0.355
**Sirt3**	0.150	0.253	-0.046	0.726	0.041	0.757	0.173	0.191
**Sirt5**	0.117	0.372	-0.016	0.902	0.177	0.176	-0.031	0.816
**Sirt6**	0.105	0.425	0.051	0.701	0.200	0.125	0.137	0.301
**Sirt7**	-0.077	0.556	0.017	0.898	-0.083	0.530	-0.022	0.871

	**Doxorubicin**		**Etoposide**		**5-Fluorouracil**		**Hydroxyurea**	
**Isoenzyme**	R	p	R	p	R	p	R	p
**HDAC1**	-0.039	0.770	0.078	0.560	-0.315	0.015	-0.119	0.369
**HDAC2**	0.150	0.252	-0.014	0.913	-0.173	0.187	-0.125	0.341
**HDAC4**	-0.103	0.439	-0.099	0.454	-0.082	0.536	0.038	0.773
**HDAC6**	-0.066	0.619	-0.079	0.550	0.193	0.140	0.018	0.891
**Sirt1**	-0.115	0.381	-0.202	0.122	-0.127	0.333	-0.016	0.906

**Sirt2**	0.030	0.819	0.051	0.701	-0.162	0.215	-0.171	0.192
**Sirt3**	-0.144	0.271	-0.151	0.248	-0.003	0.985	0.000	0.999
**Sirt5**	-0.013	0.919	-0.198	0.130	0.112	0.396	-0.055	0.677
**Sirt6**	-0.126	0.336	-0.315	0.014	0.018	0.889	0.034	0.795
**Sirt7**	-0.143	0.275	0.000	0.999	-0.135	0.302	-0.083	0.531

	**Imatinib**		**Lomustine**		**Melphalan**		**Methotrexate**	
**Isoenzyme**	R	p	R	p	R	p	R	p
**HDAC1**	-0.235	0.073	-0.149	0.259	-0.090	0.499	-0.322	0.013
**HDAC2**	0.015	0.910	-0.127	0.332	-0.055	0.675	-0.187	0.153
**HDAC4**	-0.091	0.493	-0.153	0.246	-0.125	0.347	-0.086	0.519
**HDAC6**	-0.178	0.177	-0.272	0.035	-0.203	0.120	-0.017	0.896
**Sirt1**	-0.020	0.883	-0.320	0.013	-0.161	0.220	-0.131	0.320
**Sirt2**	0.142	0.285	-0.149	0.255	-0.254	0.050	-0.071	0.587
**Sirt3**	0.102	0.444	-0.030	0.818	-0.063	0.633	-0.002	0.987
**Sirt5**	0.072	0.590	-0.121	0.359	-0.047	0.720	-0.088	0.504
**Sirt6**	-0.166	0.208	-0.209	0.109	0.038	0.776	-0.101	0.442
**Sirt7**	0.062	0.644	0.189	0.148	0.020	0.882	-0.178	0.172

	**Oxaliplatin**		**Paclitaxel**		**Thiotepa**		**Topotecan**	
**Isoenzyme**	R	p	R	p	R	p	R	p
**HDAC1**	-0.192	0.144	-0.091	0.491	-0.093	0.486	-0.171	0.196
**HDAC2**	-0.136	0.304	0.129	0.327	-0.168	0.200	-0.200	0.125
**HDAC4**	-0.086	0.516	-0.136	0.305	-0.065	0.625	-0.055	0.680
**HDAC6**	0.206	0.118	-0.118	0.371	-0.009	0.947	0.001	0.991
**Sirt1**	0.022	0.866	-0.158	0.227	-0.056	0.672	-0.090	0.492
**Sirt2**	-0.284	0.029	0.051	0.700	-0.283	0.029	-0.331	0.010
**Sirt3**	-0.048	0.716	-0.134	0.309	0.008	0.951	-0.041	0.757
**Sirt5**	0.157	0.234	-0.044	0.740	-0.046	0.729	0.050	0.704
**Sirt6**	0.095	0.474	-0.218	0.094	0.006	0.965	0.089	0.501
**Sirt7**	-0.090	0.497	-0.181	0.166	-0.066	0.615	-0.061	0.641

	**Trichostatin A**		**Vorinostat**					
**Isoenzyme**	R	p	R	p				
**HDAC1**	-0.396	0.161	-0.264	0.362				

**HDAC2**	0.308	0.285	0.278	0.336				
**HDAC4**	0.315	0.273	0.387	0.171				
**HDAC6**	-0.280	0.332	-0.031	0.916				
**Sirt1**	-0.479	0.083	-0.398	0.158				
**Sirt2**	0.020	0.946	0.015	0.959				
**Sirt3**	-0.418	0.137	-0.389	0.169				
**Sirt5**	0.179	0.539	0.106	0.718				
**Sirt6**	-0.371	0.192	-0.468	0.092				
**Sirt7**	-0.333	0.244	-0.217	0.456				

Table S8. Localization of HDAC isoenzymes across the chromosomes and corresponding reference sequence; from on UCSC Genome Browser.

**Isoenzyme**	**Localisation**	**RefSeq**
HDAC1	1p35.2-p35.1	NM_004964
HDAC2	6q21	NM_001527
HDAC4	2q37.3	NM_006037
HDAC6	Xp11.23	MN_006044
Sirt1	10q21.3	NM_012238
Sirt2	19q13.2	NM_034146
Sirt3	11p15.5	NM_001370314
Sirt5	6p24.1	NM_012241
Sirt6	19p13.3	NM_016539
Sirt7	17q25.3	NM_016538

Figure S1. Representative electropherogram showing the total protein content of the panel of 17 cancer cell lines used as internal controls in all Western blotting, as developed according to the TGX Stain Free Gels systems from Bio-Rad. Below are pre-stained protein-ladder, Page ruler ™ from Thermo Fisher #26616.

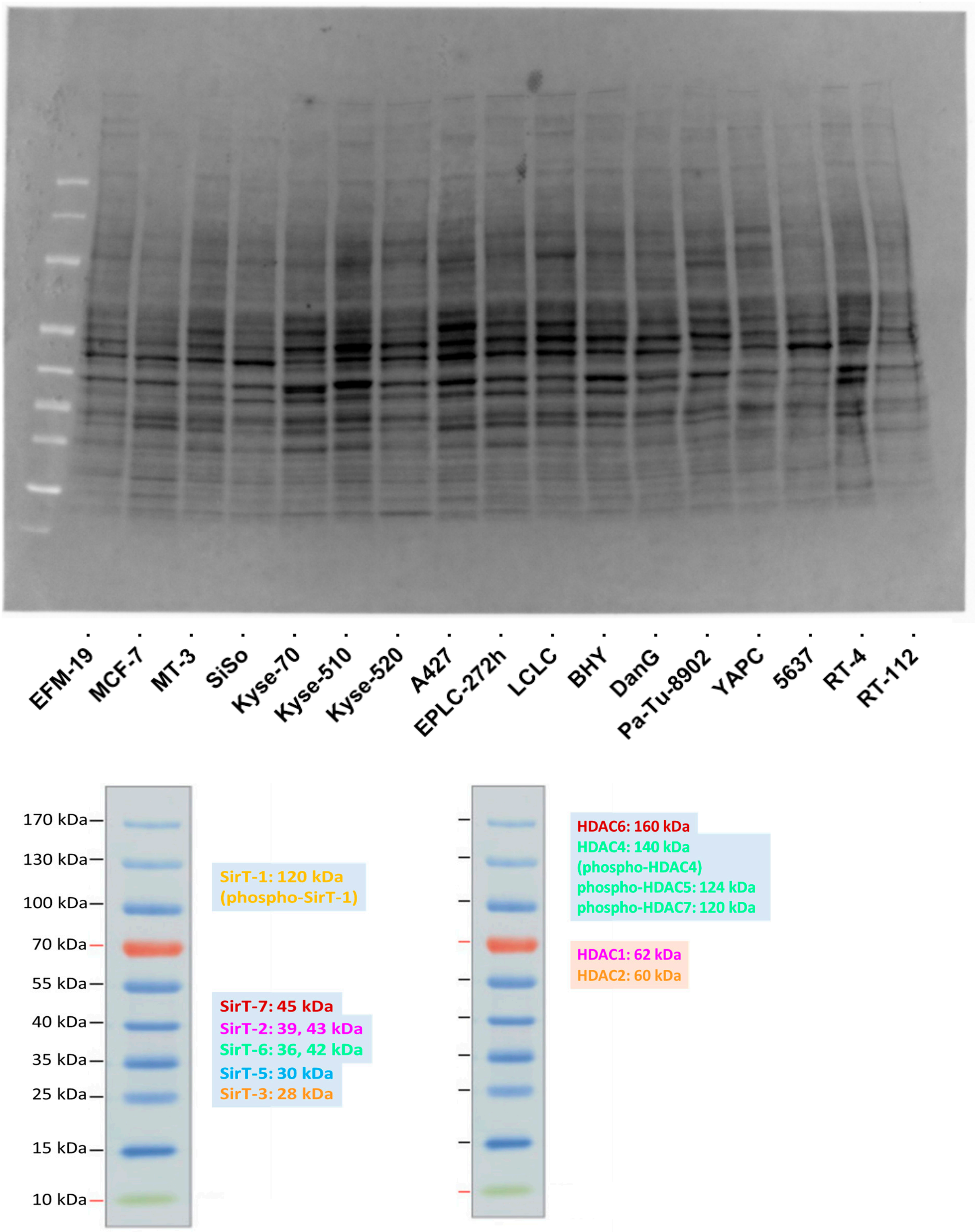

Figure S2. Western blots for the panel of 17 cancer cell lines for HDAC1, marked with antibody #5356 from Cell Signaling Technology (below: manufacture's quality control blot). Arrow indicates the band used for the analysis. The order of cell lines is shown in Figure S1.

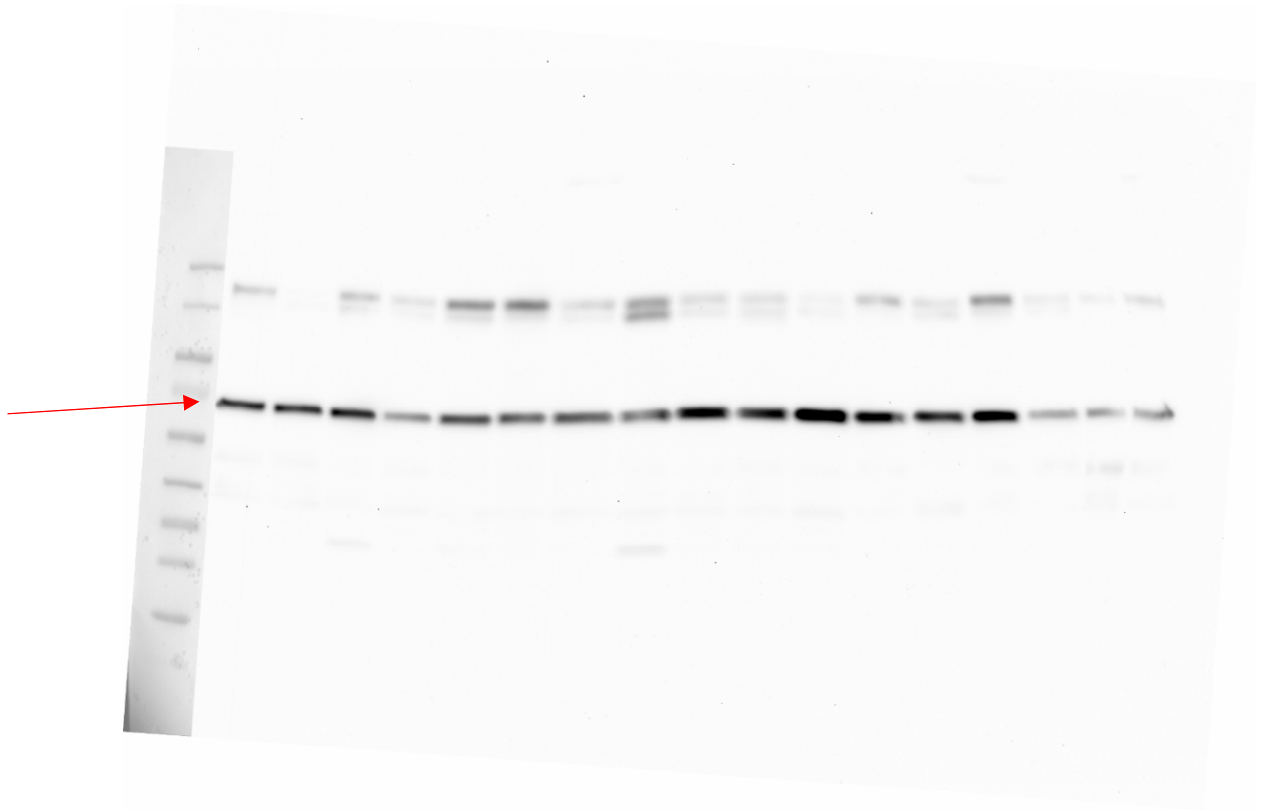

#5356

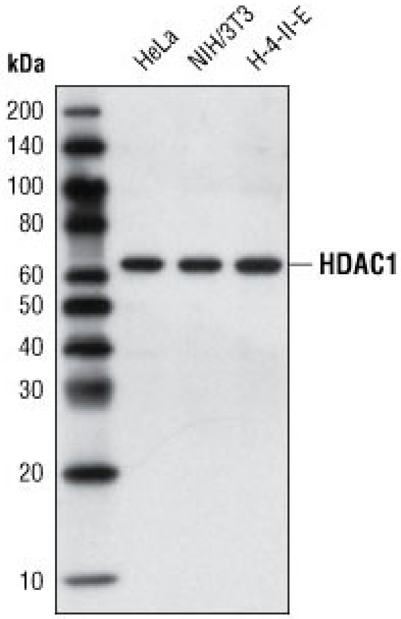

Figure S3. Western blots for the panel of 17 cancer cell lines for HDAC2, marked with antibody #5113 from Cell Signaling Technology (below: manufacture's quality control blot). Arrow indicates the band used for the analysis. The order of cell lines is shown in Figure S1.

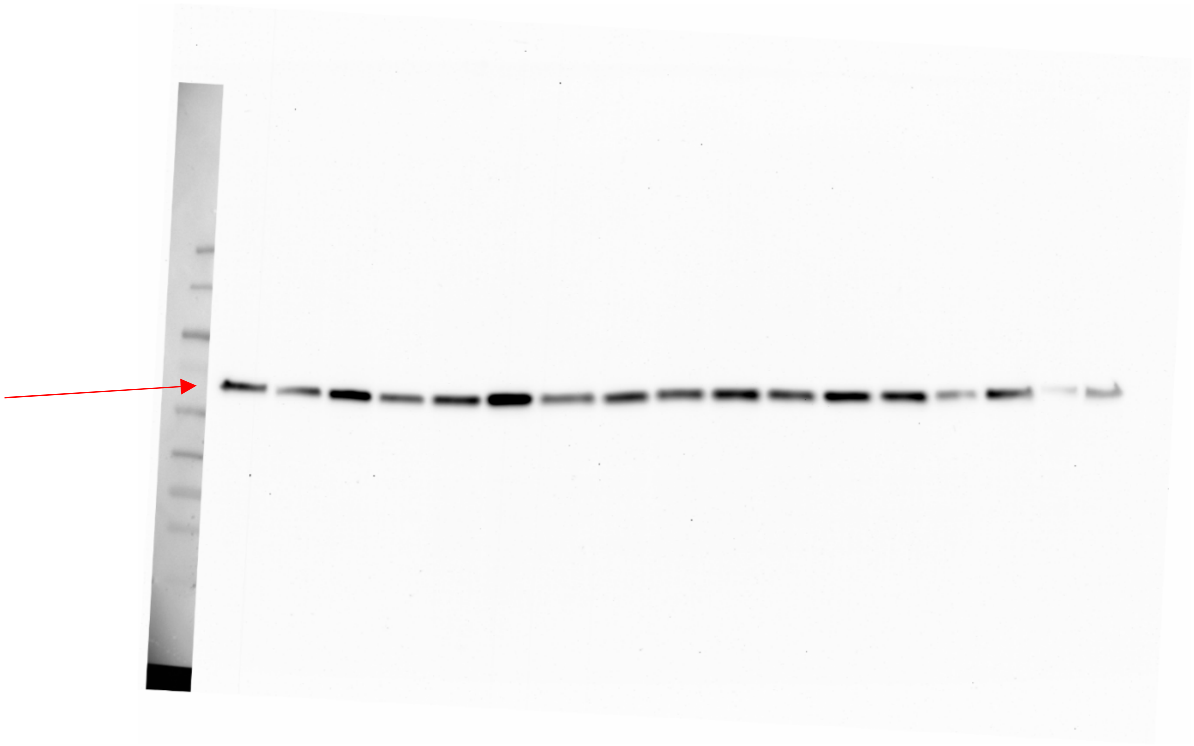

#5113

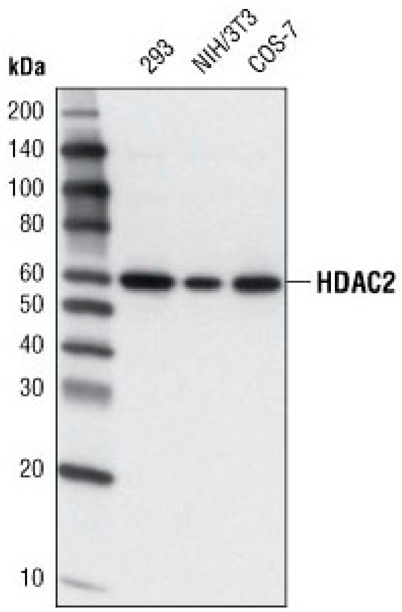

Figure S4. Western blots for the panel of 17 cancer cell lines for HDAC4, marked with antibody #7628 from Cell Signaling Technology (below: manufacture's quality control blot). Arrow indicates the band used for the analysis. The order of cell lines is shown in Figure S1.

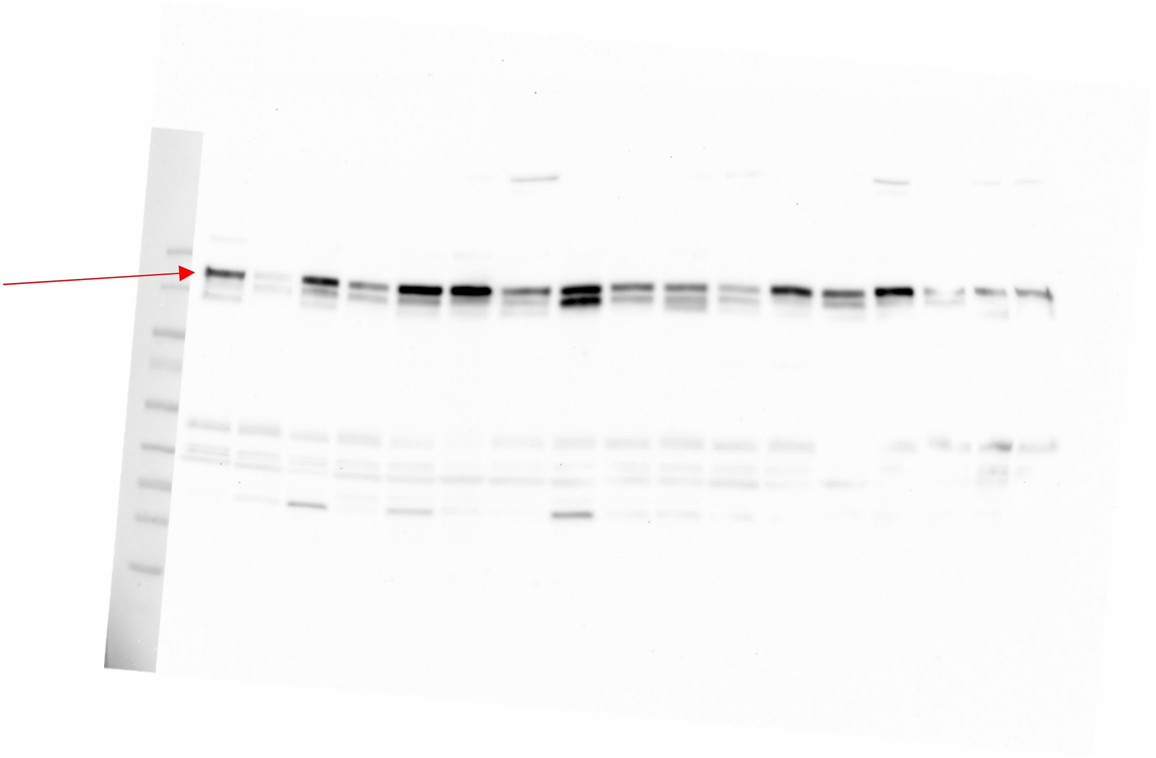

#7628

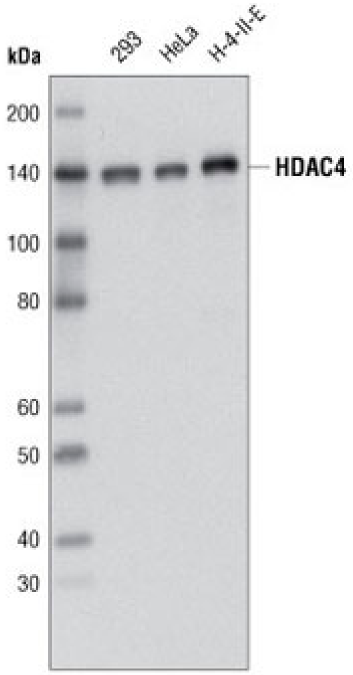

Figure S5. Western blots for the panel of 17 cancer cell lines for HDAC6, marked with antibody #7558 from Cell Signaling Technology (below: manufacture's quality control blot). Arrow indicates the band used for the analysis. The order of cell lines is shown in Figure S1.

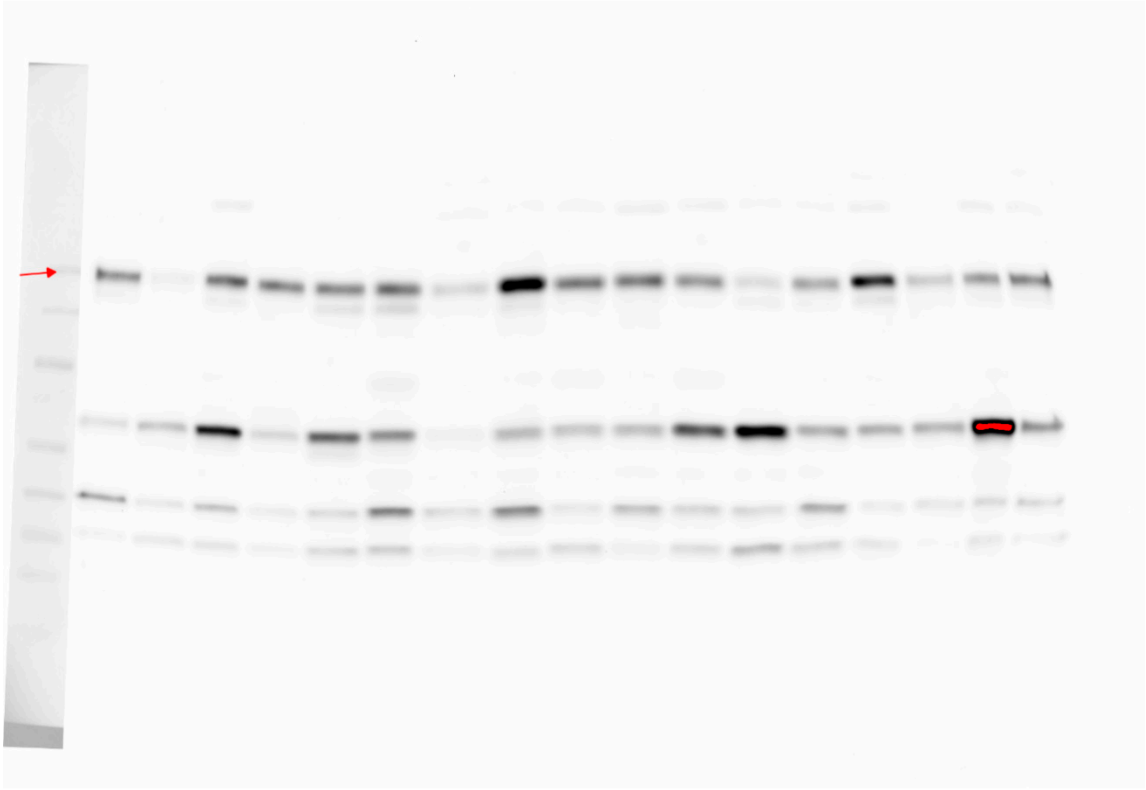

#7558

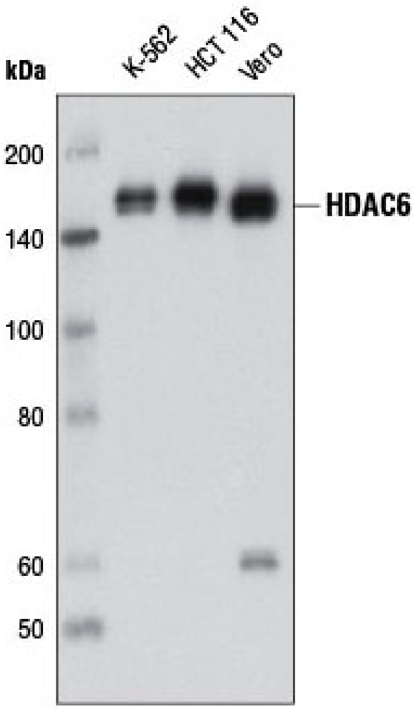

Figure S6. Western blots for the panel of 17 cancer cell lines for Sirt1, marked with antibody #9475 from Cell Signaling Technology (below: manufacture's quality control blot). Arrow indicates the band used for the analysis. The order of cell lines is shown in Figure S1.

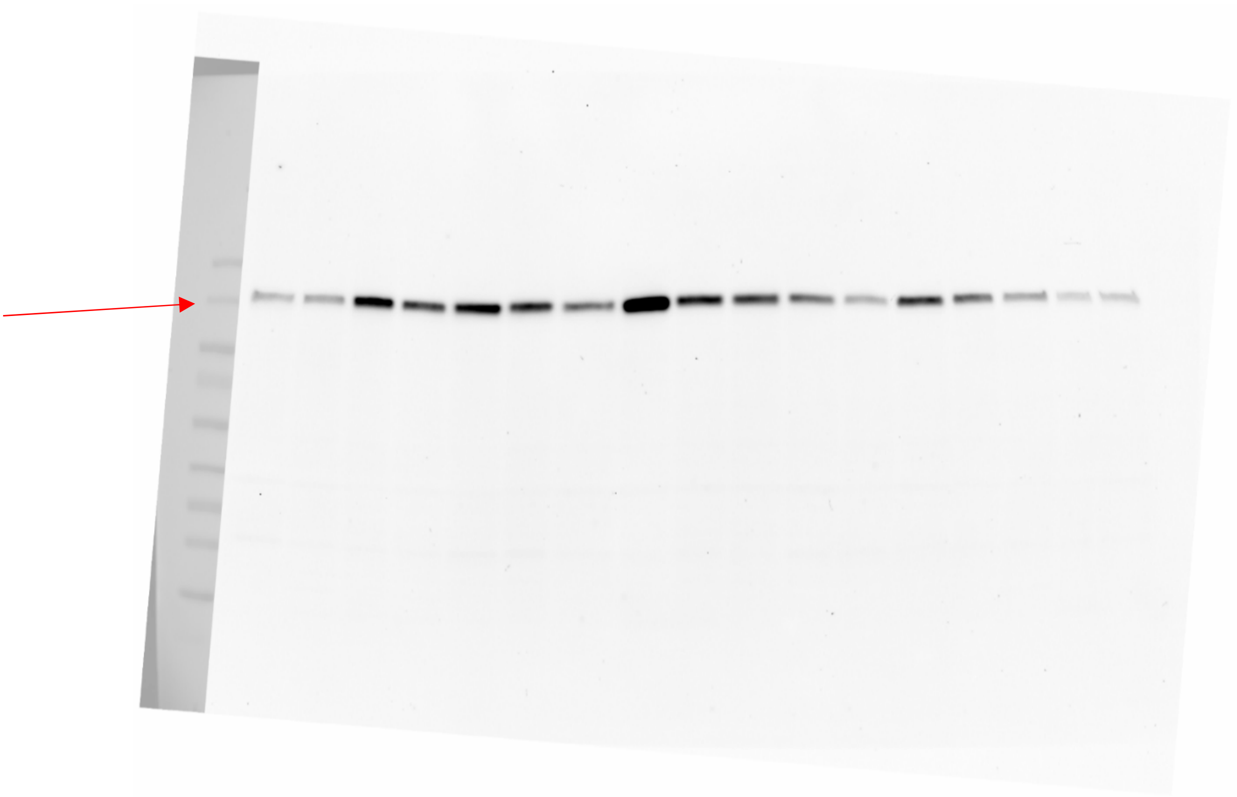

#9475

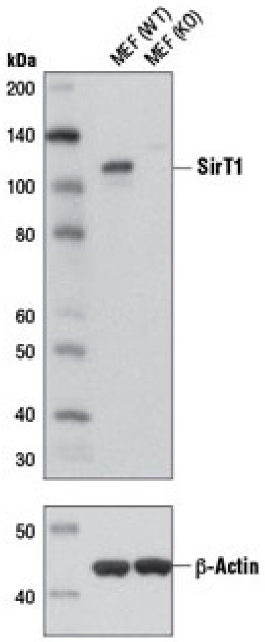

Figure S7. Western blots for the panel of 17 cancer cell lines for Sirt2, marked with antibody #12650 from Cell Signaling Technology (below: manufacture's quality control blot). Arrows indicate the bands used for the analysis. The order of cell lines is shown in Figure S1.

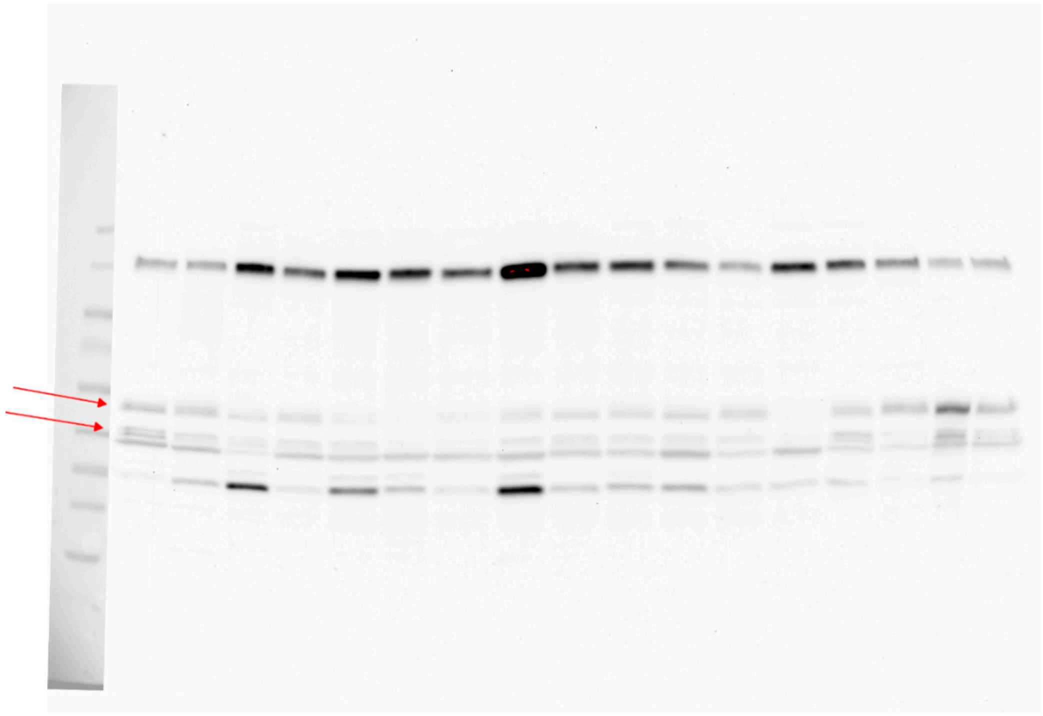

#12650

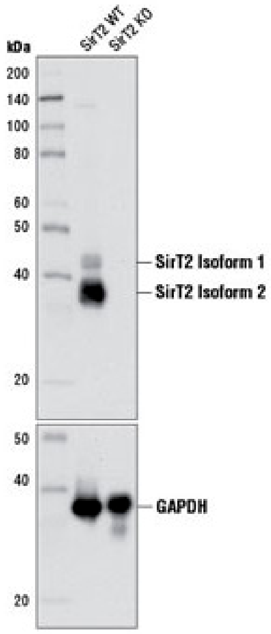

Figure S8. Western blots for the panel of 17 cancer cell lines for Sirt3, marked with antibody #5490 from Cell Signaling Technology (below: manufacture's quality control blots). Arrow indicates the band used for the analysis. The order of cell lines is shown in Figure S1.

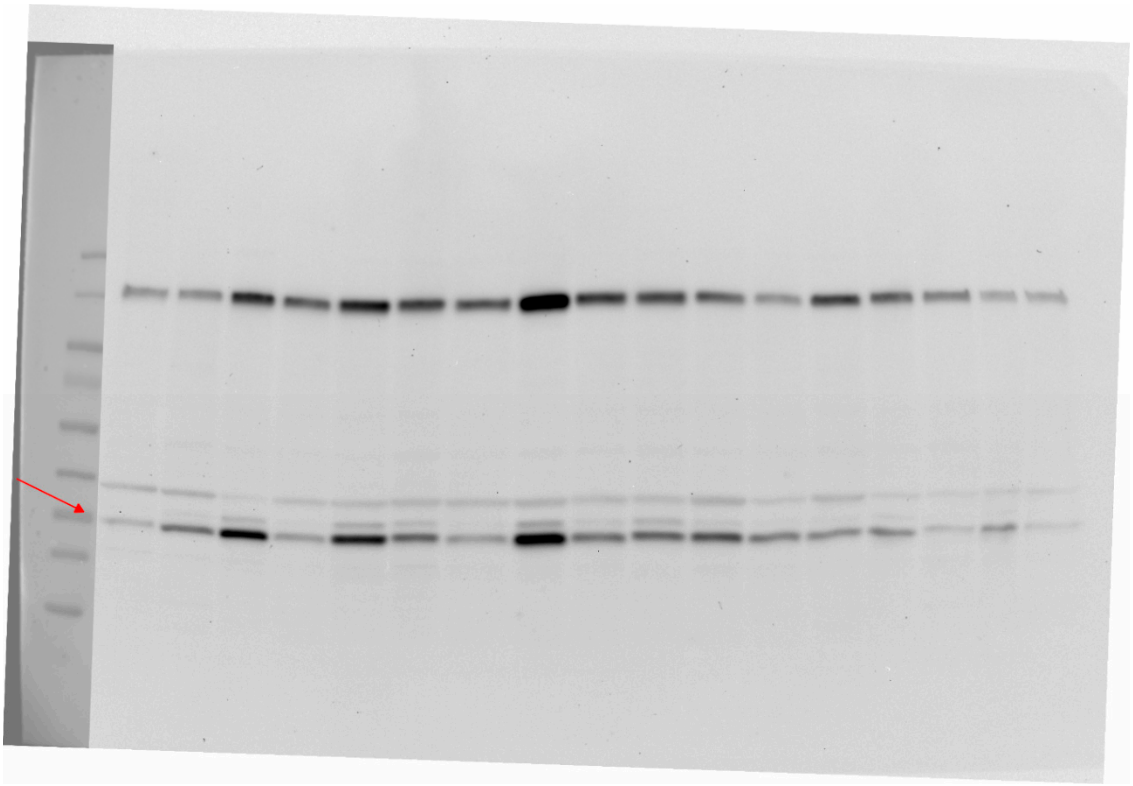

#5490

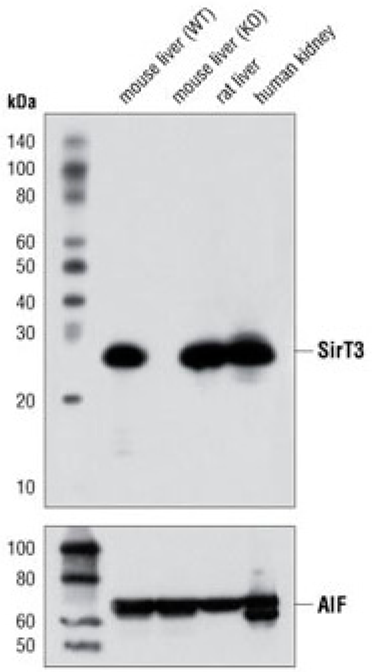

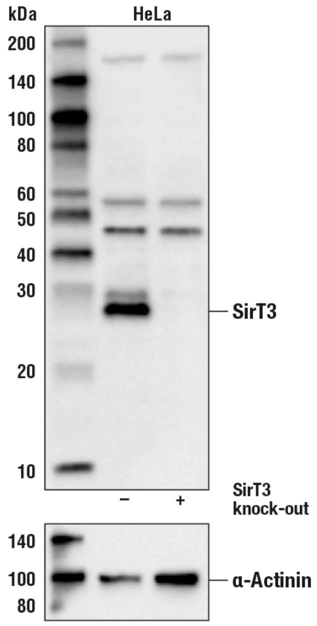

Figure S9. Western blots for the panel of 17 cancer cell lines for Sirt5, marked with antibody #8782 from Cell Signaling Technology (below: manufacture's quality control blot). Arrow indicates the band used for the analysis. The order of cell lines is shown in Figure S1.

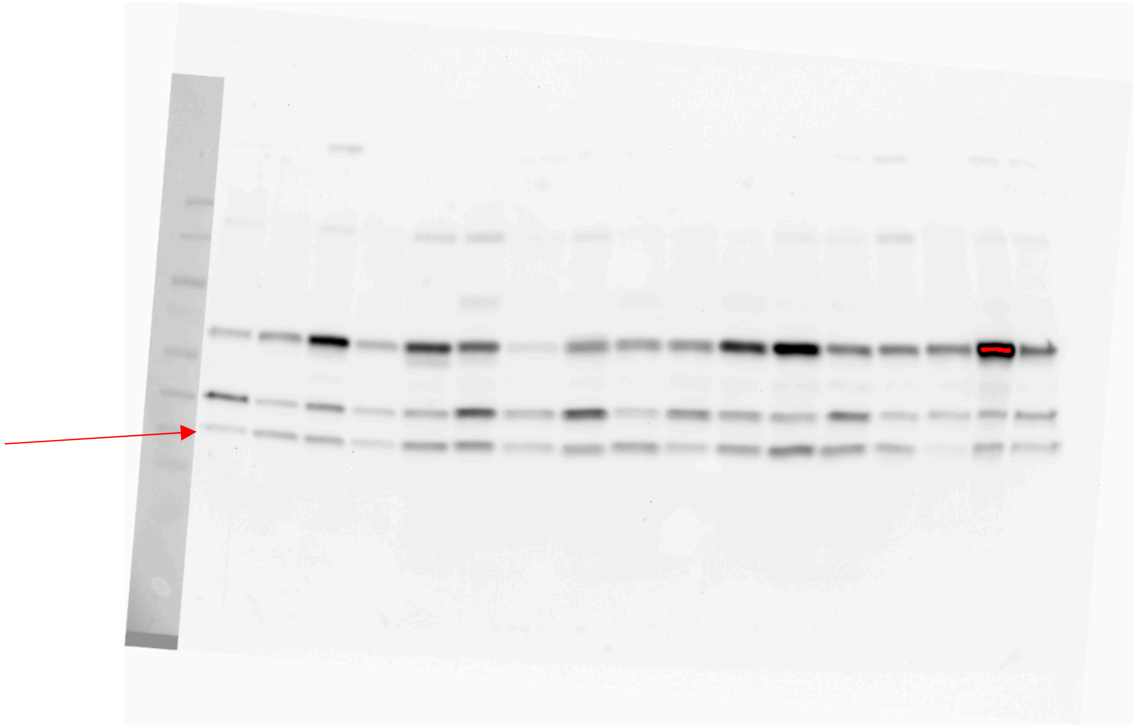

#8782

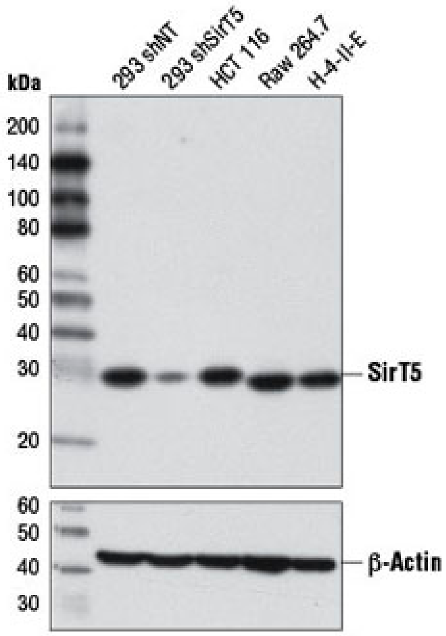

Figure S10. Western blots for the panel of 17 cancer cell lines for Sirt6, marked with antibody #12486 from Cell Signaling Technology (below: manufacture's quality control blots). Arrows indicate the bands used for the analysis. The order of cell lines is shown in Figure S1.

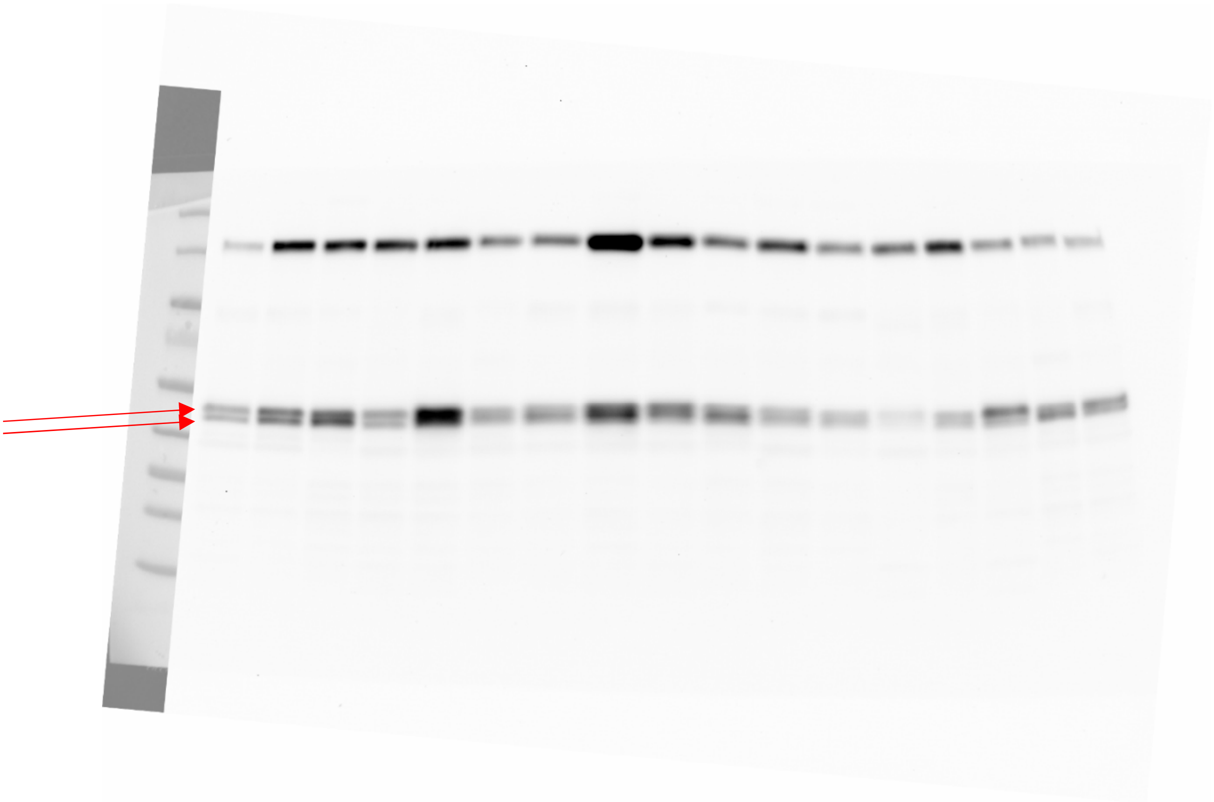

#12486

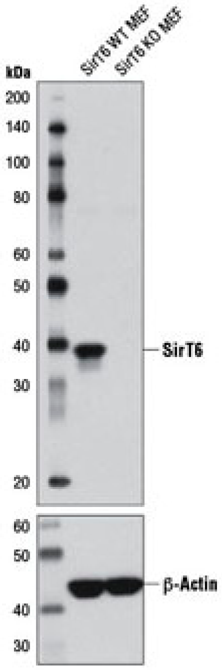

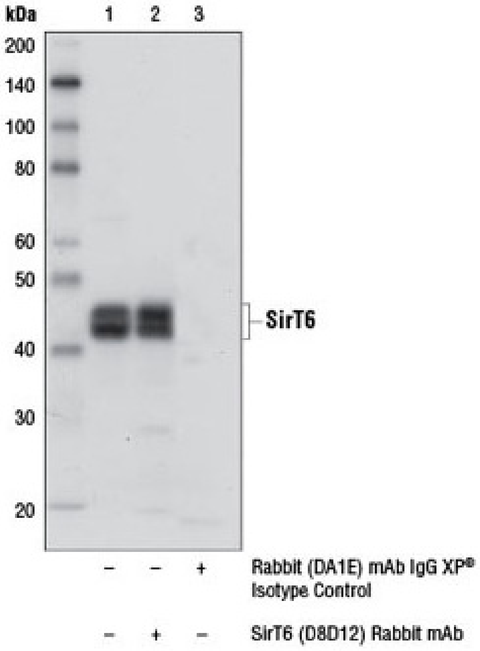

Figure S11. Western blots for the panel of 17 cancer cell lines for Sirt7, marked with antibody #5360 from Cell Signaling Technology (below: manufacture's quality control blot). Arrow indicates the band used for the analysis. The order of cell lines is shown in Figure S1.

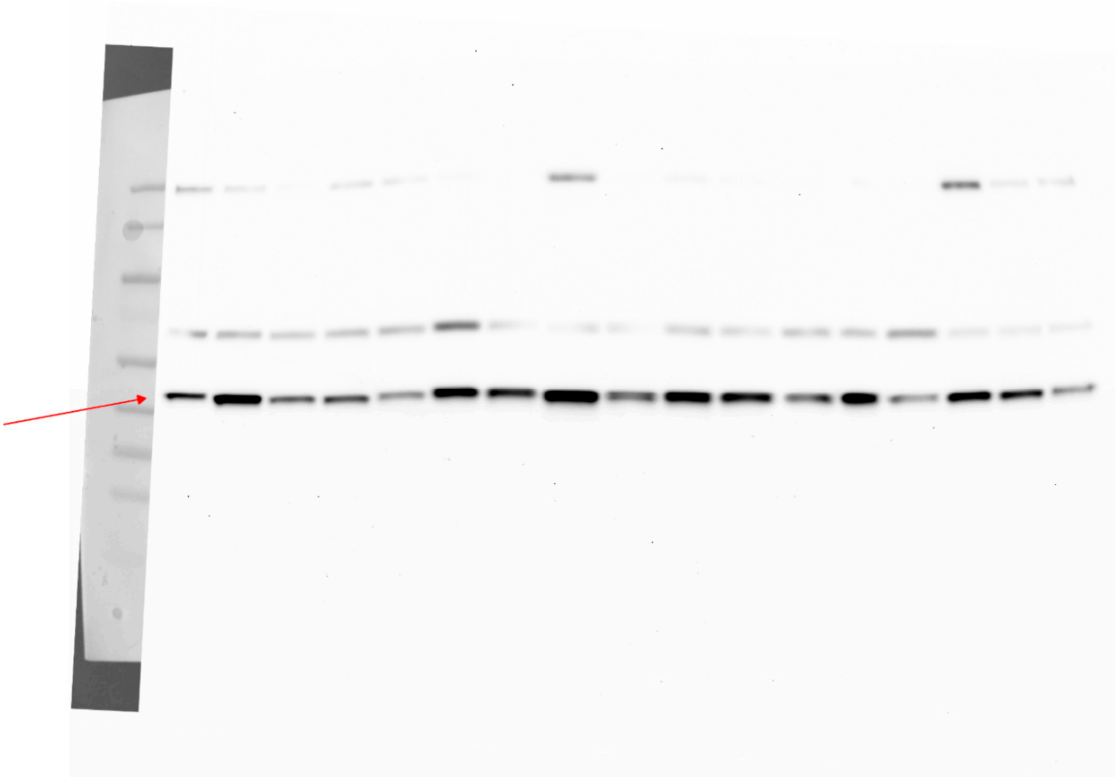

#5360

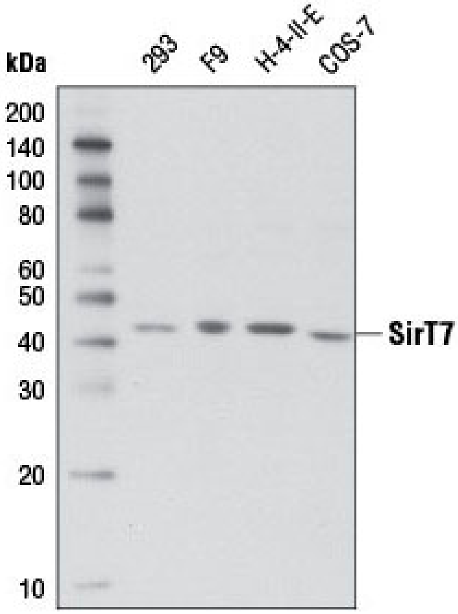

Figure S12.Univariate correlation matrix of the Spearman correlation coefficients for the expression of HDAC/Sirt isoenzyme proteins [statistics: ◆ $p<0.05$, ● not significant]. Positive correlations are depicted in blue, negative in red (see scale-bar).

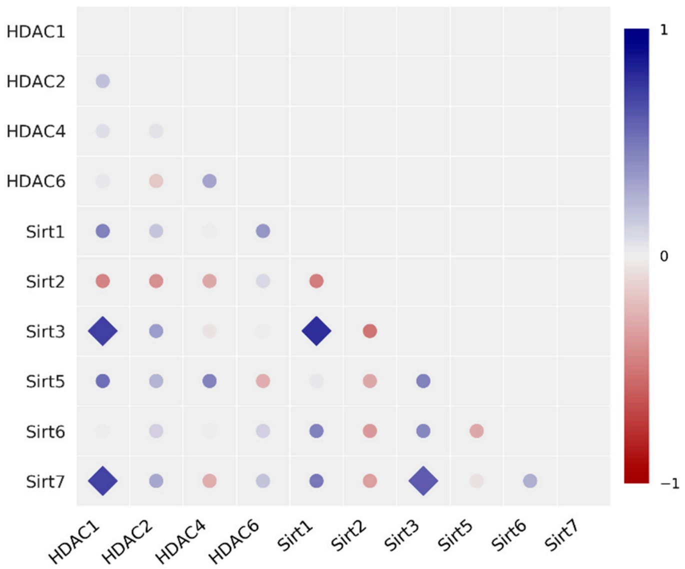

Figure S13. Univariate correlation matrix of the Spearman correlation coefficients with the NCI data for the expression of HDAC/Sirt isoenzyme mRNA [statistics: ◆ $p<0.05$, ● not significant]. Positive correlations are depicted in blue, negative in red (see scale-bar).

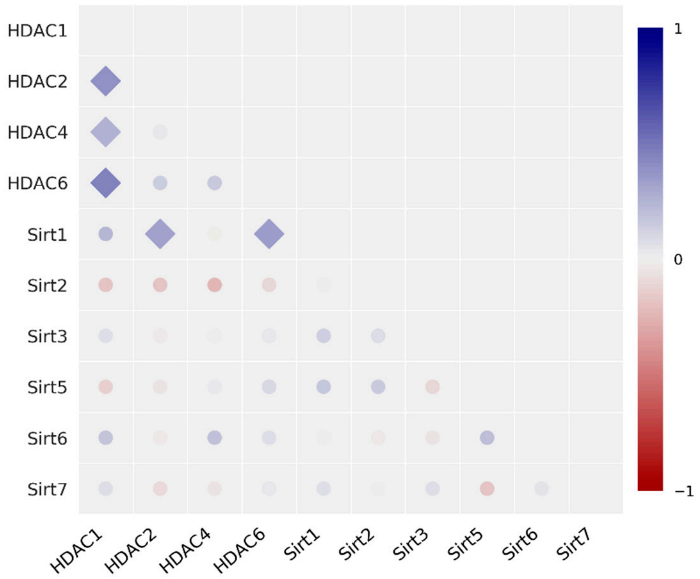

Figure S14. Univariate correlation matrix of the Spearman correlation coefficients for the expression of the HDAC/Sirt isoenzyme protein with anticancer drug potency expressed as GI_{50} values and the doubling time of cancer cells [statistics: ◆ $p<0.05$, ● not significant]. Positive correlations are depicted in blue, negative in red (see scale-bar).

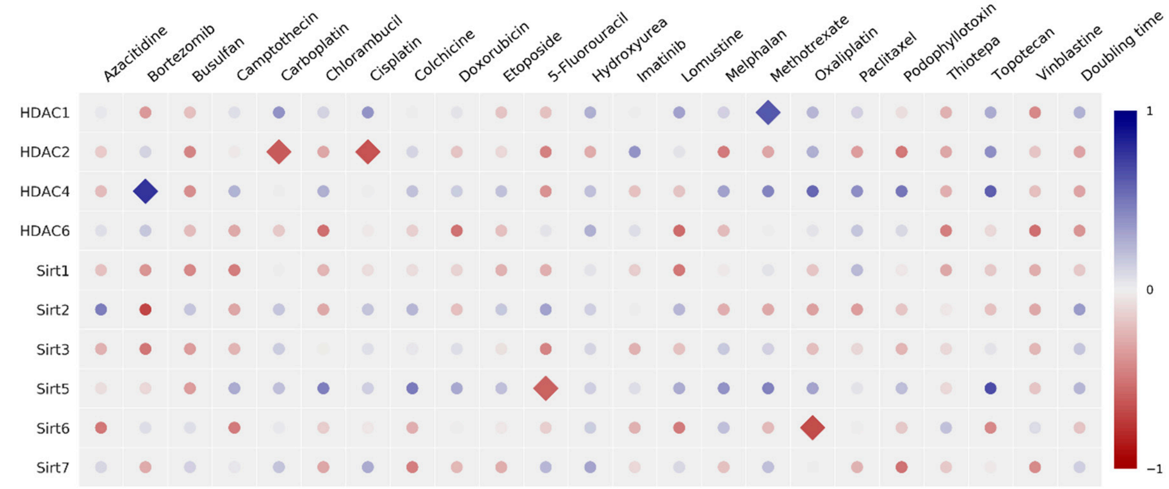

Figure S15. Univariate correlation matrix of the Spearman correlation coefficients correlation coefficients with the NCI data for the expression of the HDAC/Sirt isoenzyme mRNA with anticancer drug potency expressed as GI_{50} [statistics: ◆ $p<0.05$, ● not significant]. Positive correlations are depicted in blue, negative in red (see scale-bar).

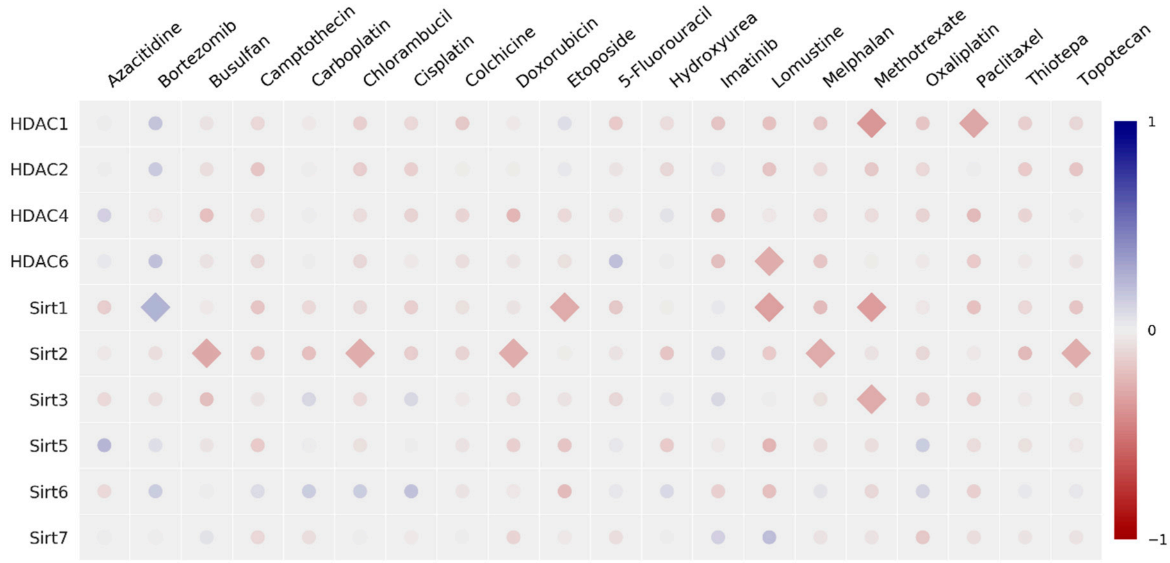

Figure S16. Univariate correlation matrix of the Pearson with FDR correction correlation for the expression of HDAC/Sirt isoenzyme proteins.

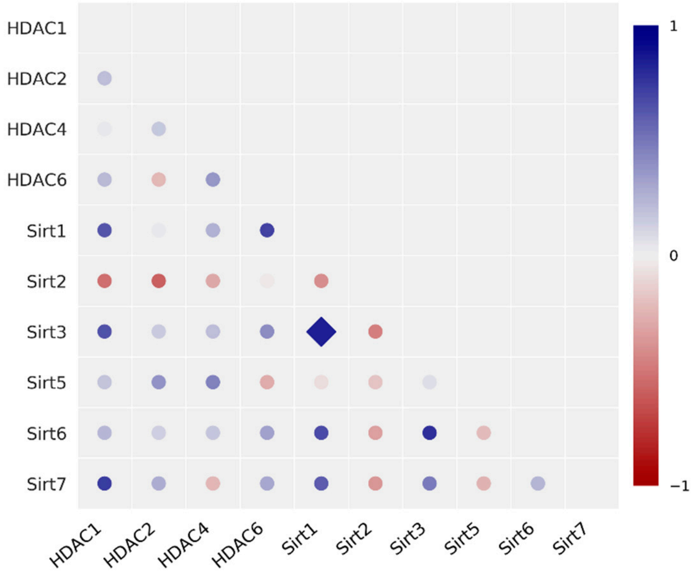

Figure S17. Univariate correlation matrix of the Pearson with FDR correction correlation with the NCI data for the expression of HDAC/Sirt isoenzyme mRNA.

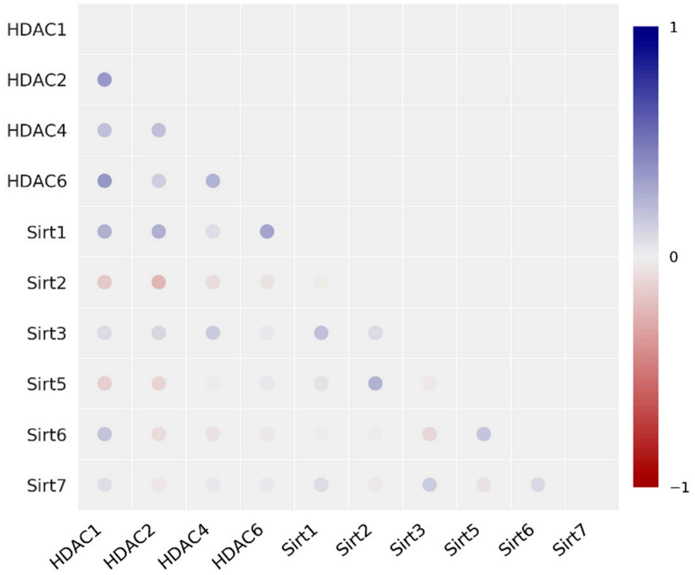

Figure S18. Univariate correlation matrix of the Pearson with FDR correction correlation for the expression of the HDAC/Sirt isoenzyme protein with anticancer drug potency expressed as GI_{50} values and the doubling time of cancer cells [statistics: ◆ $p<0.05$, ● not significant]. Positive correlations are depicted in blue, negative in red (see scale-bar).

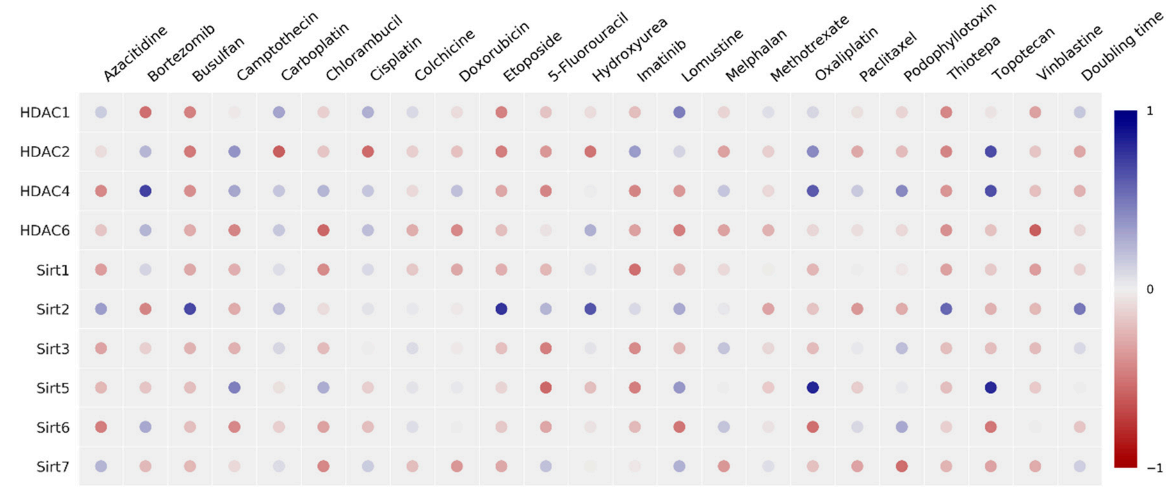

Figure S19. Univariate correlation matrix of the Pearson with FDR correction correlation for the expression of the HDAC/Sirt isoenzyme protein with anticancer drug potency expressed as GI_{50} [statistics: ◆ $p<0.05$, ● not significant]. Positive correlations are depicted in blue, negative in red (see scale-bar).

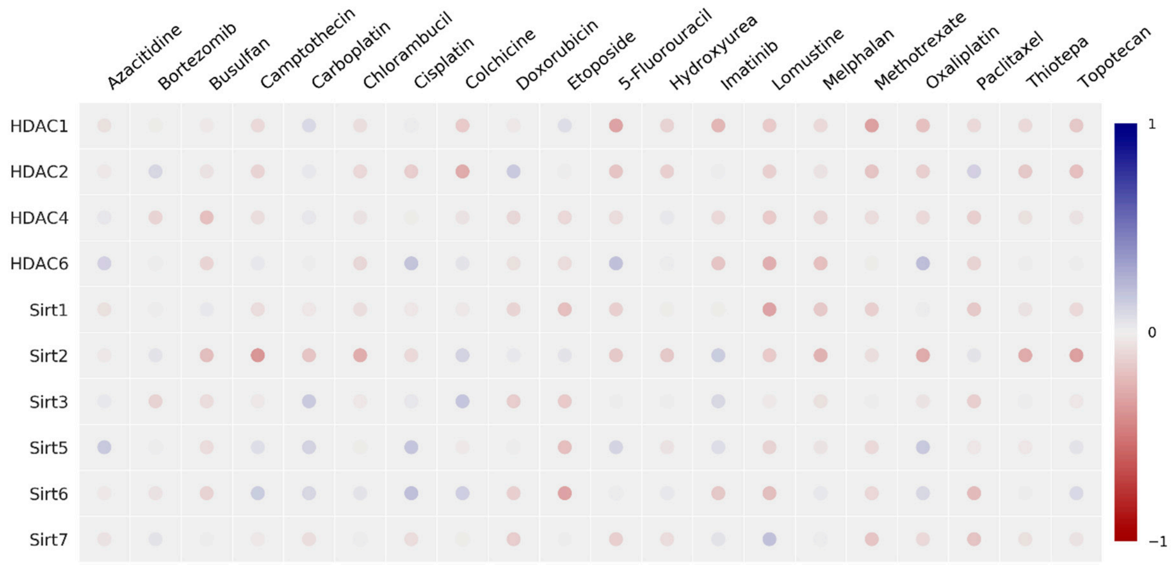

Supplement: Supplementary file 1 [file cancers-14-00187-s001.zip › cancers-1484283-supplementary.pdf]
